# Electrode Materials for Flexible Electrochromics

**DOI:** 10.3390/ijms26073260

**Published:** 2025-04-01

**Authors:** Martin Rozman, Miha Lukšič

**Affiliations:** Faculty of Chemistry and Chemical Technology, University of Ljubljana, Večna pot 113, 1000 Ljubljana, Slovenia; miha.luksic@fkkt.uni-lj.si

**Keywords:** flexible electrodes, conductive polymers, silver nanowires, metal mesh, conductive ceramics, carbon nanotubes, electrochromism, thin film, device architecture

## Abstract

Flexible electrochromic devices (ECDs) represent a distinctive category in optoelectronics, leveraging advanced materials to achieve tunable coloration under applied electric voltage. This review delves into recent advancements in electrode materials for ECDs, with a focus on silver nanowires, metal meshes, conductive polymers, carbon nanotubes, and transparent conductive ceramics. Each material is evaluated based on its manufacturing methods and integration potential. The analysis highlights the prominent role of transparent conductive ceramics and conductive polymers due to their versatility and scalability, while also addressing challenges such as environmental stability and production costs. Use of other alternative materials, such as metal meshes, carbon materials, nanowires and others, are presented here as a comparison as well. Emerging hybrid systems and advanced coating techniques are identified as promising solutions to overcome limitations regarding flexibility and durability. This review underscores the critical importance of electrode innovation in enhancing the performance, sustainability, and application scope of flexible ECDs for next-generation technologies.

## 1. Introduction

The phenomena of electrochromism, where an electrochemical device is capable of changing coloration under applied electric voltage and retaining desired coloration even after it has been disconnected from electrical power source, has been known for more than half a century [1]. Despite many improvements during their development, these types of devices never achieved widespread utilization and were shadowed by other display methods, namely liquid crystal displays (LCDs) and light-emitting diodes (LEDs). Nevertheless, there are certain applications where electrochromic devices (ECDs) excel compared to other types of displays, such as smart windows, tunable mirrors, and e-readers [2].

Multiple types of ECDs exist that use different electrochromic materials and can use different types of electrodes, such as thin film metal deposits, transparent conductive oxides, or transparent conductive polymers. These conductive polymers, such as polyethylenedioxythiophene (PEDOT) and polyethylenedioxythiophene polystyrene sulfonate (PEDOT/PSS), are invaluable in optoelectronic research since they are often the main material from which optically transparent electrodes (OTEs) are made. Polymers can be prepared in different ways, but in recent years supercritical CO_2_ drying has been shown as the most promising candidate to be used in large industrial scales [3]. ECDs can be operated by various chemical reactions or physical phenomena: redox reactions [4], ion intercalation [5], proton transfer [6], electrodeposition [7], and electrophoresis [8]. Certain modes of operation can be used in different combinations and have different time responses and coloration characteristics [9]. The most commercially known is electronic ink, which was developed in the 1970s and is now found mainly in portable e-readers. It is a type of electrophoretic screen based on colored mineral oil with dispersed metal oxide microcapsules [10]. Electrochromic materials that have gained popularity in the last two decades include transparent conductive polymers with added redox dyes (viologens) [11] for portable applications, although ceramic coated glass and thin film metal deposits coated on glass substrate are still the most widely used because glass is more stable and has better transparency.

A critical component of any ECD is its electrodes. Electrodes not only provide the necessary electrical conductivity to facilitate redox reactions but also significantly influence the device’s overall performance, durability, and scalability [12]. Various types of electrodes are used depending on the application, ranging from rigid, optically transparent electrodes for windows to flexible electrodes for wearable devices. Electrodes in ECDs serve multiple functions: they supply the electric voltage required for redox reactions, maintain the structural integrity of the device, and often contribute to its optical transparency. Conductivity, transparency, and stability are three key attributes that dictate the choice of electrode material. Transparent conductive ceramics (TCCs), such as fluorine-doped tin oxide (FTO) and indium tin oxide (ITO), have been widely used for their excellent electrical and optical properties [13]. However, their brittleness and high cost pose challenges for applications in flexible and low-cost devices.

**Figure 1:**
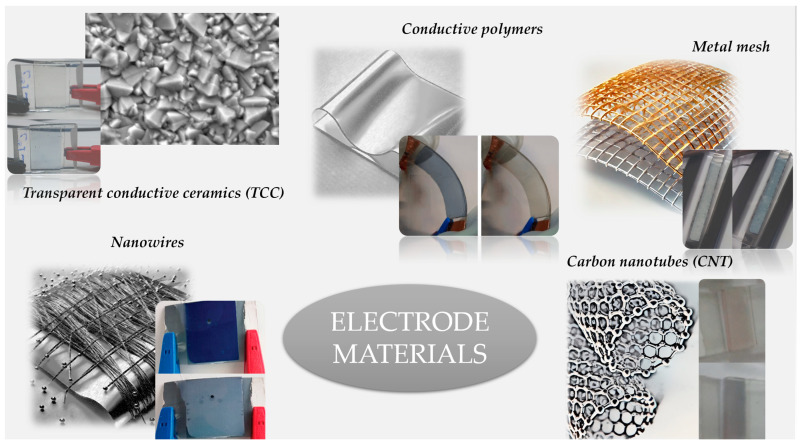
Different electrode materials and their implementation in flexible ECDs.

In this paper, we will review materials that can be used in flexible ECD construction and implementation of such materials. Most common materials in flexible ECDs are displayed in Figure 1.

## 2. Flexible Electrochromic Devices Architecture

While flexible electrochromic and electronic devices hold immense promise, several challenges must be addressed. This includes mechanical durability, where repeated bending and stretching can degrade device performance [14,15]. Hybrid materials and reinforced composites, for example those combining conductive polymers with metal mesh, are being developed to enhance mechanical stability [16,17]. In addition to that, materials that are used in construction must be stable enough to survive bending, stretching attempts, as well as humidity and UV exposure. The performance of flexible ECDs and other electronic devices heavily relies on the transparent conductive electrodes. Materials like silver nanowires (AgNWs) [18], carbon nanotubes (CNTs) [19], graphene [20], and conductive polymers [21] provide the necessary balance of conductivity, transparency, and flexibility. For example, AgNWs exhibit exceptional electrical conductivity with sheet resistances as low as 15 Ω/sq at 90% transmittance [22], making them a leading choice for flexible transparent conductive electrodes (TCEs). While electrode material is important, it should not be overlooked that an important factor represents the flexibility of substrate along with its ability to accept adhesion of electrode material. Flexible substrates such as polyethylene terephthalate (PET) and polyimide (PI) offer mechanical stability and transparency [23]. Some of the flexible substrates are compatible even with transparent conductive ceramics (TCCs), with a good example being indium-doped tin oxide (ITO), which enables TCCs to be used in these applications, although with certain limitations.

**Figure 2:**
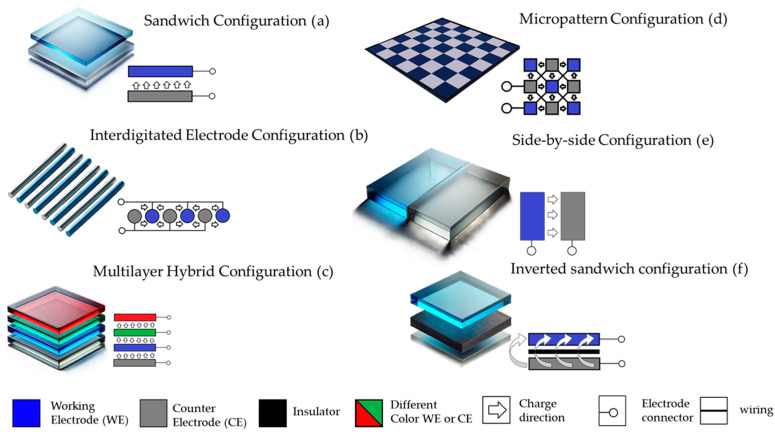
Examples of different architectures, showing emplacement of electrodes and its charge flow.

The architecture of electrochromic devices (ECDs) significantly influences their performance, durability, and scalability, and has become a focal point of research and development in advanced materials science [24,25,26,27]. Various architectures have been developed, each tailored to specific applications or optimized for distinct performance metrics. This section provides a detailed overview of the most prominent and emerging architectures in ECDs, detailing their structures, functionalities, and challenges (Figure 2).

- Sandwich Configuration: The sandwich ECD architecture represents the most conventional design in ECDs [28,29]. In this configuration, the device comprises sequentially stacked layers, including a transparent conductive electrode, an electrochromic layer, an electrolyte, and a counter electrode (Figure 2a). The transparent conductive electrode, often fabricated from indium tin oxide (ITO) or silver nanowires (AgNWs), serves as the base layer, ensuring electrical connectivity and optical transparency. The electrochromic layer, typically made of materials like tungsten oxide (WO_3_) [30] or PEDOT/PSS [31], modulates light transmission through reversible redox reactions. A solid, gel, or liquid electrolyte facilitates ion transport, while the counter electrode, such as nickel oxide (NiO) [32], balances ionic charges during device operation. This architecture is particularly suited for applications requiring high optical contrast and uniform coloration, such as smart windows and e-paper displays. Flexible and stretchable ECDs are designed to maintain functionality under mechanical deformation, including bending, stretching, and twisting. These devices utilize elastic substrates such as polydimethylsiloxane (PDMS) [33,34] or thermoplastic polyurethane (TPU) [35] in conjunction with flexible conductive electrodes like carbon nanotubes (CNTs) [36,37,38], graphene, or AgNWs [33,34]. The electrochromic materials are often embedded within or coated onto these substrates to ensure mechanical durability. This architecture is particularly suited for wearable electronics and biomedical applications, where flexibility and lightweight construction are essential. Despite its mechanical advantages, the lifespan of such devices may decrease under extreme deformation conditions [39]. Additionally, achieving a balance between flexibility and high optical modulation remains a challenge [40,41].
- Interdigitated Electrode Configuration: The interdigitated configuration employs a patterned arrangement of electrochromic and counter-electrode materials in an alternating “finger-like” structure (Figure 2b) [42]. The gaps between the interdigitated fingers are filled with an electrolyte, enabling efficient ion transport. This design offers several advantages, including faster switching speeds and precise control over coloration due to the short ionic pathways between the electrodes. High-resolution displays and adaptive optical systems are among the primary applications for this architecture. The fabrication of interdigitated configurations requires advanced techniques such as lithography or high-precision printing, making it a costly option for large-scale production. Furthermore, maintaining uniformity across the patterned electrodes poses significant challenges.
- Multilayer Hybrid Configuration: This architecture combines multiple functional materials and layers to achieve superior durability, optical modulation, and environmental resistance (Figure 2c) [43,44,45]. Devices constructed in this architecture often integrate inorganic and organic materials, such as WO_3_ combined with conductive polymers like PEDOT/PSS, to leverage the benefits of both material types [46,47,48]. This architecture is widely used in advanced displays and energy-efficient windows, where high durability and performance are paramount. However, the increased complexity of the fabrication process and the higher cost of materials may hinder widespread adoption.
- Micropatterned Configuration: Micropatterning involves segmenting the electrochromic material into discrete regions or “pixels,” enabling localized control over optical modulation (Figure 2d) [49,50]. Advanced lithographic techniques or printing methods are used to create high-resolution patterns, making this architecture suitable for e-paper displays and adaptive optics. While micropatterned architectures offer unparalleled design flexibility, they require precise fabrication processes that can be expensive and time-intensive. Uniformity across patterns and scalability remains a significant hurdle for widespread application.
- Side-by-Side Electrode Configuration: In the side-by-side configuration, both the electrochromic and counter electrodes are positioned laterally on the same substrate (Figure 2e) [51]. A thin electrolyte layer bridges the gap between the electrodes, facilitating ionic movement [52,53]. This architecture is particularly advantageous for thin and lightweight devices, as it eliminates the need for multiple stacked layers, reducing overall device thickness. While this design simplifies device structure, it inherently limits the coloration area due to the lateral positioning of the electrodes as the counter electrode often does not have any color change. Additionally, the longer ionic pathways between adjacent electrodes can lead to slower switching speeds compared to other architectures [54,55].
- Reverse Sandwich Configuration: This alternative, also known as “inverted sandwich” architecture, is a lesser-used configuration in which the roles of certain layers are reversed, or non-transparent materials such as metal foils replace conventional transparent electrodes (Figure 2f) [56,57]. In this design, the electrochromic material is directly deposited onto the metal foil, which acts as both the electrode and the substrate. The electrolyte layer is subsequently applied, followed by a counter electrode positioned on the opposite side. This architecture is ideal for non-transparent applications, including smart mirrors and adaptive thermal shields, where optical transparency is not required. Stainless steel or aluminum foils often serve as substrates, providing exceptional mechanical durability and chemical stability [57,58]. Despite its simplified fabrication and robust mechanical properties, this architecture is not useful for see-through devices and may face challenges in achieving high optical contrast.

The diverse architectures of ECDs cater to a wide range of applications, from flexible wearables to advanced displays and energy-efficient systems. Each design presents unique advantages and challenges, and the choice of architecture depends on specific performance requirements and intended use cases. Different designs are presented in Figure 2. As fabrication techniques and material science continue to advance, novel architectures are expected to emerge, further expanding the potential of electrochromic devices in modern technology.

## 3. Electrodes in Flexible ECDs

### 3.1. Silver Nanowires

Silver nanowires (AgNWs) [18,33,34,59,60] are a prominent choice for electrodes in electrochromic devices (ECDs) due to their exceptional electrical conductivity, high optical transparency, and mechanical flexibility. Unlike traditional transparent conductive oxides (TCOs), such as fluorine-doped tin oxide (FTO), AgNWs are suitable for flexible and stretchable applications, making them particularly attractive for next-generation wearable and foldable devices. Recent advancements in the synthesis and processing of AgNWs have further expanded their potential in scalable manufacturing and high-performance ECDs. For comparison, bulk silver exhibits a resistivity of approximately 1.59 µΩ cm^−1^ [61], one of the lowest among metals. AgNWs, due to their high aspect ratio, can achieve conductivities close to bulk silver [62], especially when surface oxidation is minimized and junctions between nanowires are optimized. AgNW networks can be engineered to balance conductivity and optical transparency. For instance, a study reported achieving a sheet resistance of 15.6 Ω/sq at a transmittance of 90% [22] by reducing the thickness of the polyvinylpyrrolidone (PVP) layer on the nanowires, which improved contact between them. In general, the AgNWs show sheet resistance between 6.5 and 50 Ω/sq and transmittance between 80 and 92.5% [63]. The majority of the reported papers describe preparation of AgNW via a polyol process, showing relatively high transmittance (90–92.5%), with variable sheet resistance, depending on the thickness of prepared layers (8.6–45 Ω/sq). The sheet resistance and transmittance data shown in this paper are collected from the cited literature. Table 1 summarizes selected works where AgNWs with different sheet resistance and transmittance values were used.

#### 3.1.1. Manufacturing Methods of AgNW

The synthesis of AgNWs typically involves solution-based methods, with the polyol process being the most widely used [22,64,67]. In this method, silver nitrate (AgNO_3_) acts as the precursor, and ethylene glycol serves as both the reducing agent and solvent. Polyvinylpyrrolidone (PVP) is used as a capping agent to control the growth and prevent aggregation of the nanowires. This process produces uniform nanowires with high aspect ratios, which are critical for achieving low sheet resistance and high transparency. After synthesis, the AgNWs are dispersed in solvents for deposition onto substrates with one of the following techniques:

1. Spray Coating: A scalable method where the AgNW solution is sprayed onto the substrate, offering uniform coverage and compatibility with large-area devices [68].
2. Spin Coating: Used for smaller-scale applications, this method ensures uniform thin films by spinning the substrate at high speeds. This method is commonly used in laboratories, as it provides precise and repeatable preparation of samples [69].
3. Surface plasma treatment: Surface plasma treatment is a method that is generally used as a post-treatment to enhance the properties of AgNWs, particularly their electrical conductivity and adhesion to substrates. The material is first prepared by using one of the more common procedures and involves exposing AgNWs to a plasma, which helps to remove contaminants, oxide layers, or organic coatings. By reducing the insulating layer (e.g., PVP), plasma treatment lowers the contact resistance between nanowires, enhancing network conductivity [70].
4. Electron beam lithography (EBL): This method uses a focused beam of electrons to directly write custom patterns. By focusing a beam of electrons onto a substrate coated with an electron-sensitive resist, EBL can directly write patterns without the need for masks, achieving resolutions below 10 nanometers [66].

The choice of deposition method depends on the intended application, substrate type, and desired electrode properties.

#### 3.1.2. Chemical Stability of AgNW

AgNWs are inherently prone to oxidation when exposed to air or moisture, leading to a loss of electrical conductivity and device performance over time. To mitigate this issue, researchers have developed several protective strategies. These include coating AgNWs with conductive polymers; for example, PEDOT/PSS improves their chemical stability and flexibility, and preserves their electrical properties [71,72]. Another option is by applying transparent, inert encapsulation layers, such as thin films of graphene oxide [73] or silicon dioxide [74], that creates a barrier against environmental degradation. Last option is alloying, where other metals like gold or copper are incorporated into the nanowires, which reduces their susceptibility to oxidation without significantly affecting their conductivity. These strategies extend the operational lifetime of AgNW-based electrodes, making them viable for long-term applications.

#### 3.1.3. Durability of AgNW

AgNWs exhibit excellent mechanical properties, retaining their conductivity under repeated bending, stretching, and twisting [75,76]. This makes them ideal for flexible and wearable devices. Durability tests have shown that AgNW electrodes can withstand exposure to bending, with low degradation of electrical performance [77,78]. However, challenges remain in improving their thermal stability, as elevated temperatures can lead to junction breakdown and performance loss. To enhance durability, researchers have explored several methods such as nanowire welding [79], where by photonic sintering or thermal annealing, the resistance of the junction is reduced. as Also, embedding AgNWs in flexible polymer matrices prevents wire displacement and adds mechanical strength.

#### 3.1.4. Device Construction Capabilities of AgNW

AgNW-based electrodes are highly suitable for transparent and flexible ECDs due to their low sheet resistance and high optical transmittance [59,69]. These properties make them competitive alternatives to TCOs for large-area and foldable applications [26]. Examples of AgNW integration in ECDs include wearable displays and foldable electronics, where their flexibility and mechanical resilience comes into play.

AgNWs represent a promising material for next-generation ECDs, offering unique advantages in flexibility, transparency, and electrical conductivity. However, addressing challenges such as chemical stability, thermal robustness, and large-scale uniformity is crucial for their widespread adoption. Future research will likely focus on hybrid systems that combine AgNWs with other materials, such as graphene or conductive polymers, to overcome these limitations and unlock their full potential in electrochromic applications.

### 3.2. Metal Meshes

Metal meshes have emerged as a robust and scalable option for electrodes in electrochromic devices (ECDs) [80]. These conductive grids, typically composed of metals such as silver [81], copper [82], steel [83], or gold [84], offer a unique combination of electrical conductivity, mechanical durability, and optical transparency. Unlike TCOs, metal meshes maintain excellent performance under mechanical stress [85,86], making them ideal for applications in flexible and wearable ECDs. Their scalability and cost-effectiveness further enhance their appeal for large-area electrochromic applications such as smart windows and adaptive displays. Sheet resistance is generally low, usually measuring few ohms per square, with transmittance varying depending on width, spacing between wires, and the metal used. The resistance also heavily depends on the type of material chosen [61], where typically, highly conductive metals such as copper and silver are most preferred. Due to better chemical resistance, silver and other precious metals are usually the material of choice [87,88] as they prevent possible side reactions or potential corrosion of electrodes as well as certain types of stainless steel [89] and specialty alloys [90,91]. Transmittance is usually in the range of 70–80%, with the additional property of electromagnetic shielding, which is due to the Faraday cage effect. It must be noted that thickness of the wires, along with mesh pattern, can have a substantial effect on both transmittance and sheet resistance. For instance, the paper reported by Li et al. [92] presents gold nanomesh with a width of 100–175 nm, reporting it has a reasonably low sheet resistance of 16.5 Ω/sq for 175 nm width mesh. McGehee et al. [93] presented steel (type 316) mesh with a fiber thickness of 35 µm. This type of chromium–nickel steel is commonly used in electrochemical and battery applications, as it offers reasonably high chemical resistance. This steel mesh had better characteristics, obtaining higher transmittance (87.3%) compared to gold nanomesh (65%). As individual fibers in mesh are solid objects, the transmittance values are often determined by the gaps in the mesh, while the wire thickness dictates sheet resistance. Therefore, it is possible that the values are dictated in principle by design rather than the material itself. The best values were reported by Chen et al. [94], who showed excellent sheet resistance (0.18 Ω/sq), and good transmittance (85.5%) obtained by Cu mesh with individual fibers measuring between 1.1 and 1.9 µm, which implies that good design and proper material selection are important in metal mesh preparation. It must also be noted that sheet resistance is often difficult to determine reliably, as many of the meshes do not consist of straight-line fibers but rather of interconnected strains. Table 2 summarizes selected works where various metal meshes with different sheet resistance and transmittance values were used.

#### 3.2.1. Manufacturing Methods of Metal Meshes

Metal meshes are fabricated using a variety of advanced techniques that allow precise control over their structure and dimensions, as well as depending on the metals or alloys used in the manufacturing process [80,97,98]. It is also one of the oldest and most researched methods for the manufacture of semi-transparent electrodes [99]. The choice of manufacturing method depends on the desired application, required resolution, and scalability needs.

1. Wire Knitting: Knitted wire mesh consists of a metal wire strand knitted into a mesh structure in very much the same way as thread is knitted into fabric [100].
2. Photolithography: This method involves patterning a photosensitive layer on a metal-coated substrate using UV light. The unexposed areas are then etched away, leaving behind a fine mesh pattern. Photolithography provides high precision and is widely used for small-scale applications where fine resolution is critical [98].
3. Nanoimprint Lithography: Nanoimprinting in general employs a mold or stamp to pattern the metal layer on a substrate. The specific method that is used for metal mesh production is nanoimprinting by capillary forces [101], which utilizes a metal-containing ink or precursor solution that is introduced by capillary action into the cavities between the mold and the substrate. Upon subsequent drying and annealing, well-defined metal mesh patterns are formed. This technique facilitates controlled deposition at nanoscale resolution that is compatible with flexible substrates. It enables manufacture of smaller devices or parts of devices, which can be used to manufacture a large number of devices or assemble the imprints in larger devices [102]. This cost-effective technique is suitable for large-scale production and enables the creation of nanoscale grid patterns that enhance optical transparency [103].
4. Roll-to-Roll Processing: This high-throughput manufacturing method involves depositing and patterning metal films on flexible substrates in a continuous manner. Roll-to-roll processing is particularly advantageous for producing large-area metal meshes at reduced costs [104,105].
5. Direct Printing: Techniques such as inkjet printing and aerosol jet printing allow the direct deposition of metal inks onto substrates [106]. These methods enable rapid prototyping and are compatible with various substrate types, including flexible polymers [107,108]. Recently, 3D printing has become an attractive method to prepare electrodes as well [109].

**Figure 3:**
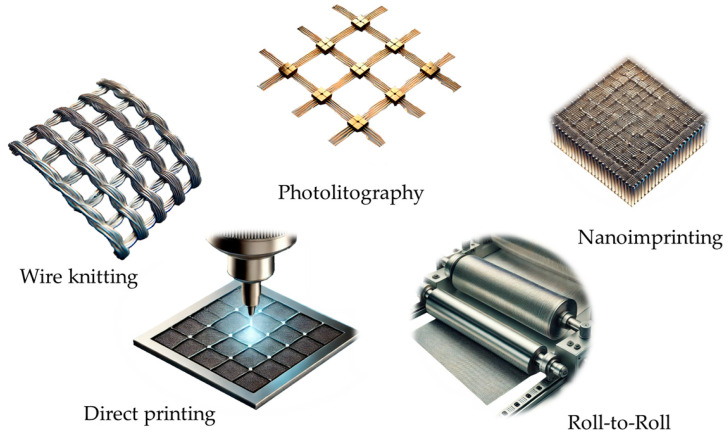
Manufacturing methods of common metal mesh electrodes.

Different Manufacturing methods are presented in Figure 3.

#### 3.2.2. Chemical Stability of Metal Meshes

Metal meshes generally exhibit excellent stability under normal operating conditions, comparable to the bulk metals and alloys from which they are made [16]. However, their susceptibility to corrosion and oxidation can affect long-term performance, especially in environments with high humidity or exposure to corrosive agents. Strategies to enhance chemical stability include applying protective coatings such as graphene or metal oxides which create a barrier against oxidation and chemical degradation [17,110,111]. While this tactic prevents corrosion, it can be in many cases unsuitable, due to high resistance of anticorrosion coatings, which renders the performance [112]. Another option is the alloying of multiple materials, especially precious metals such as gold–silver [113] or silver–palladium [114] coatings, which enhances resistance to environmental factors without compromising electrical conductivity.

#### 3.2.3. Durability of Metal Meshes

Metal meshes are renowned for their mechanical robustness, maintaining conductivity under repeated bending, stretching, and twisting [115,116,117], which makes them particularly well-suited for flexible and wearable devices. Key factors contributing to their durability include high tensile strength, allowing meshes to withstand significant mechanical stress without structural failure. In addition, integrating the mesh with flexible substrates such as PET [118] or PDMS [119], as well as fine-tuning the mesh pattern, enhances mechanical stability and prevents cracking during deformation.

#### 3.2.4. Device Construction Capabilities of Metal Meshes

Metal meshes are versatile electrodes that can be tailored for various electrochromic applications, ranging from rigid displays to flexible and foldable devices [26,80]. Microscale grid pattern devices have been shown to achieve good results and were proposed to be used in smart windows as well as bendable and wearable electronics, enabling innovative applications in adaptive clothing and curved displays. Similarly to AgNW [120], metal meshes could be easily scaled with industry-friendly methods, such as roll-to-roll processing, being able to be produced at relatively low cost.

Advances in manufacturing techniques and protective strategies have addressed many of their limitations, such as corrosion and cost. Future research should focus on optimizing mesh patterns for further improved transparency and developing hybrid systems that integrate metal meshes with other advanced materials like conductive polymers or graphene.

### 3.3. Conductive Polymers

**Figure 4:**
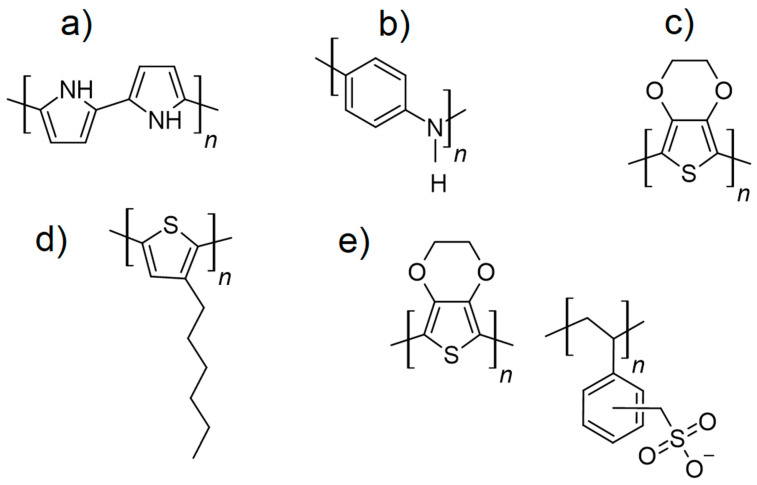
Structures of common conductive polymers, showing polypyrrole (PPy) (**a**), polyaniline (PANI) (**b**), poly(3,4-ethylenedioxythiophene) (PEDOT) (**c**), poly(3-hexylthiophene) (P3HT) (**d**), and poly(3,4-ethylenedioxythiophene)/polystyrene Sulfonate (PEDOT/PSS) (**e**).

Conductive polymers are organic materials with intrinsic electrical conductivity, making them a versatile choice for electrodes in electrochromic devices (ECDs) [21,24,121,122,123]. These are by far the most investigated materials for use with flexible electronics. Transparent conductive polymers (TCPs) are a unique class of materials that combine electrical conductivity with optical transparency, enabling their use in a variety of optoelectronic applications. These materials are lightweight, flexible, and can be processed using solution-based techniques, making them highly attractive for applications requiring large-area and flexible electrodes. The following section highlights key transparent conductive polymers and their properties. Figure 4 represents the structure of the most commonly used conductive polymers.

#### 3.3.1. Poly(3,4-ethylenedioxythiophene)/Polystyrene Sulfonate (PEDOT/PSS)

PEDOT/PSS is the most widely used TCP due to its excellent electrical conductivity, high optical transparency, and mechanical flexibility [3,124]. It is composed of PEDOT [31], the conducting component, and PSS, which acts as a stabilizing agent. PEDOT/PSS films can achieve sheet resistances and transmittance comparable to traditional TCOs like indium tin oxide (ITO), especially after post-treatment with sulfuric acid [125], DMSO [126], or ethylene glycol [127] to enhance conductivity. Applications of PEDOT/PSS include electrochromic devices (ECDs), organic photovoltaics, OLEDs, and touchscreens, making it a versatile material in optoelectronics.

#### 3.3.2. Polyaniline (PANI)

PANI is a TCP that can exhibit transparency in its emeraldine salt form [128,129]. It is known for its tunable electrical properties, achieved through doping or changes in its oxidation state. PANI’s applications include antistatic coatings, flexible transparent electrodes, and electrochromic films. However, its transparency and environmental stability are moderate compared to PEDOT/PSS, which limits its broader adoption.

#### 3.3.3. Polypyrrole (PPy)

PPy is another TCP recognized for its stability and tunable electrical properties [130,131,132]. Transparent PPy films can be fabricated, though their optical transmittance and conductivity are generally lower than PEDOT/PSS. PPy mostly finds applications in sensors, actuators, and electrochromic devices where moderate conductivity and transparency are sufficient [133,134].

#### 3.3.4. Polythiophenes (PTs)

PTs, particularly poly(3-hexylthiophene) (P3HT), represent an important class of conductive polymers. These materials exhibit good conductivity and transparency in thin film form [135,136]. PTs are often used in organic photovoltaics and transparent electronics, though their performance is typically lower than PEDOT/PSS in terms of optical and electrical properties [137].

#### 3.3.5. Emerging TCPs and Specialized Materials

- Polyfluorenes: Known for their optical properties, polyfluorenes can be doped to achieve conductivity [138]. They are primarily used in research applications for light-emitting devices and flexible electronics.
- Poly(ethylene dioxythiophene-co-dioxythiophene) (PEDOT/DOT): A derivative of PEDOT, PEDOT/DOT provides enhanced transparency and electrical conductivity, making it suitable for advanced applications in flexible displays and wearable electronics [139].

In many cases, conductive polymers are combined with other materials to form hybrid systems in order to obtain more favorable results [137]. An excellent example is PEDOT/PSS, which is often prepared as a hybrid material. By default, PEDOT/PSS often has high sheet resistance measuring from 29 to 70 Ω/sq for a multilayer system to more than 1.6 kΩ/sq for a single layer system [140]. This can be improved by additives or other TCE materials, such as acid treatment [141] or application of polymer to metal mesh [142]. PEDOT/PSS is not the only polymer that can be improved this way, as other polymers such as PANI can be improved in a similar manner, reducing sheet resistance from 1.1 k Ω/sq [143] to less than 100 Ω/sq [144].

Table 3 summarizes some of the works of various conductive polymers with different sheet resistance and transmittance values.

#### 3.3.6. Manufacturing Methods of Conductive Polymers

TCPs can be processed using various techniques to produce thin films and composites and have a variety of methods on how to be prepared. Key manufacturing methods for TCPs along with their advantages are presented in Table 4.

- Poly(3,4-ethylenedioxythiophene) Polystyrene Sulfonate (PEDOT/PSS)

There are two distinctive ways of preparing PEDOT/PSS:

- Solution Processing: The material is dissolved in water or alcohol-based solvents to create a colloidal dispersion [141,147]. Further thin film preparation techniques include spin coating, spray coating, dip coating, and inkjet printing, which allow precise deposition on substrates of various sizes and shapes [148,149,150,151].
- Electropolymerization: This method involves the electrochemical deposition of PEDOT on an electrode from a monomer solution [152]. This method enables controlled film thickness and uniformity.

In both cases, the prepared thin films are often treated with sulfuric acid, DMSO, or ethylene glycol to enhance electrical conductivity by reducing the insulating PSS component [126,127]. The material manufacturing methods offer high scalability with solution processing methods, compatible with large-area and flexible substrates as well as high conductivity and optical transparency. The downside is that the manufacturing has to be performed in a low humidity or water-free environment as the polymer is highly sensitive to water exposure. Additional challenges are present during the post-treatment process and UV exposure, which complicates large-scale production.

- Polyaniline (PANI) and Polypyrrole (PPy)

The main method is chemical oxidative polymerization: Aniline or pyrrole monomers are polymerized using oxidants (e.g., ammonium persulfate) in acidic solutions, producing conducting emeraldine salt, while pyrrole monomers are polymerized in the presence of oxidants like ferric chloride, yielding conductive films or powders [153,154]. Thin films are then cast or deposited on substrates. Both of them can be polymerized electrochemically on conductive substrates, enabling precise film thickness control. PANI can be blended with flexible polymers or conductive fillers to enhance mechanical properties and/or conductivity. While both of the materials offer high environmental stability and are suitable for making composites or mixing with other conductive materials, they have relatively low conductivity and transparency. Both of the polymers can be improved by using carbon-based fillers [144,155], co-polymers [156], metal oxides [157], or metal nanoparticles [158] that increase conductivity. PPy has around 30% transmittance compared to PEDOT/PSS (around 80–90%) and also lacks mechanical stability compared to the latter.

- Polythiophenes (PTs)

The synthesis of polythiophenes (PTs) involves several methods. The first is chemical oxidative polymerization [159], where thiophene monomers are polymerized using oxidants like ferric chloride (FeCl_3_) or ammonium persulfate (APS). This method produces conductive polythiophene films or powders. The process is highly scalable and can be carried out in solution or directly on substrates, offering flexibility in processing. However, the resulting polythiophenes may have moderate molecular weights and some residual impurities [160]. The next method is electrochemical polymerization: this technique involves the application of an electric potential to a conductive substrate immersed in a solution of thiophene monomers and electrolytes [161,162]. Polymerization occurs directly on the electrode, resulting in films with precise control over thickness, morphology, and doping levels. While this method is ideal for research and precision applications, scalability remains a challenge for industrial use. Using catalysts such as palladium or nickel, thiophene monomers are linked in a process called transition metal catalyzed polymerization [163,164]. This method produces high molecular weight polythiophenes with well-defined structures, such as poly(3-hexylthiophene) (P3HT) [165]. These derivatives are widely used in solar cells and OLEDs [166,167]. However, the reliance on expensive catalysts and stringent reaction conditions limits cost-effectiveness. Another method is to enable solution-based deposition techniques like spin coating, drop casting, or inkjet printing, where thiophene derivatives such as P3HT are synthesized with alkyl side chains to improve solubility in organic solvents [168]. This method is well-suited for large-area film formation, particularly in flexible electronic applications. In general, polythiophenes are valued for their high conductivity, environmental stability, and mechanical flexibility. Functional derivatives, such as P3HT, provide tunable electronic properties and compatibility with scalable solution processing techniques. However, pristine polythiophene has moderate electrical conductivity and optical transparency compared to materials like PEDOT/PSS. Additionally, the need for complex doping and expensive catalysts can pose economic challenges for some manufacturing methods.

#### 3.3.7. Chemical Stability and Durability of Conductive Polymers

The manufacturing of transparent conductive polymers involves a variety of methods, each tailored to the specific properties and applications of the material. While PEDOT/PSS leads in terms of commercial viability and performance, materials like PANI, PPy, and PT offer unique advantages for niche applications [121]. Overcoming challenges in scalability, environmental stability, and uniformity will be critical to advancing the use of TCPs in next-generation optoelectronic devices. While conductive polymers offer excellent processability and conductivity, their chemical stability under environmental stressors such as moisture, UV light, and oxygen remains a significant challenge [169].

Conductive polymers exhibit great mechanical flexibility, making them ideal for applications in wearable and foldable devices. Durability tests have demonstrated their ability to maintain conductivity under repeated bending and stretching. Key factors influencing durability include film thickness, where thinner films are more flexible but may suffer from reduced electrical conductivity. Balancing thickness and performance is critical. One possible modification is also embedding conductive polymers into flexible substrates, such as thermoplastic polyurethane (TPU) [35], which enhances mechanical stability and prevents cracking. This of course usually results in lower electrical conductivity so a precise optimization is necessary.

Among TCPs, PEDOT/PSS stands out as the most practical choice for commercial applications due to its superior balance of conductivity, transparency, and environmental stability [170]. PANI and PPy offer niche advantages in tunability and specific functionalities, while materials like polythiophenes and polyfluorenes remain under active research for next-generation applications [131,135,156]. Transparent conductive polymers continue to evolve, driven by the demand for sustainable, flexible, and high-performance materials in emerging technologies. The integration of these polymers into hybrid systems and the optimization of their properties through chemical modifications promise to expand their applicability across a broader range of optoelectronic devices.

Advances in manufacturing techniques, chemical stabilization, and hybrid systems have addressed many of their limitations, such as environmental sensitivity, durability, and scalability, yet there are still challenges that prevent its use in some more specialized applications, such as electrochemical sensors [169]. Future research should focus on further enhancing their electrical conductivity and durability, particularly under extreme environmental conditions, to unlock their full potential in flexible and transparent ECDs.

### 3.4. Carbon Nanotubes

Carbon nanotubes (CNTs) are one-dimensional nanostructures composed of graphene sheets rolled into cylindrical shapes. They exhibit exceptional electrical conductivity, mechanical strength, and thermal stability, making them an attractive material for electrode applications in electrochromic devices (ECDs) [36]. CNTs are categorized into single-walled (SWCNTs) [171] and multi-walled (MWCNTs) [172] varieties, each offering unique advantages for ECD integration [19]. Their high aspect ratio, coupled with excellent flexibility, enables CNT-based electrodes to meet the demands of transparent, flexible, and stretchable devices. In general, CNT films have emerged as promising materials for transparent conductive electrodes due to their good electrical and optical properties that puts them between metal meshes and conductive polymers [26]. They have comparable transmittance to conductive polymers, while maintaining lower sheet resistance. In addition, CNTs are reasonably simple to prepare and since carbon is not critical material, reasonably accessible from the industry standpoint. Table 5 summarizes some of the works where various CNTs with different sheet resistance and transmittance values were used.

#### 3.4.1. Manufacturing Methods of CNTs

The synthesis of CNTs involves several advanced techniques, each with distinct advantages and challenges. Chemical vapor deposition (CVD) is the most widely used method, where hydrocarbon gases such as methane or ethylene are decomposed on a metal catalyst at high temperatures, yielding CNTs with controllable structures and diameters [19,173,176]. This process, however, often results in traces of catalyst being left over in prepared material. Another approach is the arc discharge method [177], an older but cost-effective technique that produces CNTs by vaporizing graphite electrodes in a helium atmosphere; however, this method typically results in lower purity compared to CVD. Laser ablation is another technique [178], utilizing a high-energy laser to vaporize a graphite target mixed with a catalyst, producing CNTs with uniform diameters, though this method is expensive and limited in scalability. After synthesis, CNTs are sometimes dispersed in solvents and deposited onto substrates using processes like spin coating, spray coating, or vacuum filtration [179,180]. Functionalization or the addition of surfactants is commonly employed to improve dispersion quality and achieve uniform film formation [181].

#### 3.4.2. Chemical Stability and Durability of CNTs

CNTs exhibit excellent intrinsic chemical stability due to their strong carbon–carbon bonds; however, challenges arise during their integration into ECDs. One primary issue is aggregation and defects, as CNTs tend to aggregate, which reduces their effective conductivity and optical transparency [182]. This problem can be mitigated through functionalization or blending with conductive polymers [183,184]. Environmental sensitivity is another concern, as exposure to oxidative environments or high humidity can degrade CNT-based films. Coatings or composite structures are often employed to enhance stability under harsh conditions. Additionally, surface functionalization with carboxyl or amine groups improves CNT compatibility with substrates and other materials, mitigating chemical degradation and ensuring robust performance [185,186]. Beyond their chemical properties, CNT electrodes are renowned for their mechanical robustness, retaining conductivity and structural integrity under mechanical stress. This durability is attributed to their high tensile strength, which is significantly greater than that of most materials, enabling them to withstand repeated bending, stretching, and twisting. Embedding CNTs in flexible polymers such as thermoplastic polyurethane (TPU) further enhances mechanical stability while maintaining flexibility [173]. In general, CNT-based electrodes demonstrate resilience to repeated mechanical cycles, retaining over 95% of their conductivity even after thousands of bending cycles, highlighting their suitability for applications requiring durability and flexibility [187].

Carbon nanotubes offer exceptional electrical conductivity, mechanical flexibility, and environmental stability. While challenges such as dispersion and scalability remain, recent advances in functionalization, hybrid systems, and large-scale processing techniques have addressed many of these issues.

### 3.5. Transparent Conductive Ceramics

Transparent conductive ceramics (TCCs) are by far the most widely used as TCEs in a variety of devices, including electrochromic devices (ECDs) [188]. Known for their excellent optical transparency, electrical conductivity, and environmental stability, TCCs have become a cornerstone in applications such as displays [189], photovoltaics [190], and smart windows [191]. This section explores key transparent conductive ceramics, their properties, advantages, and challenges.

#### 3.5.1. Tin Oxide-Based Electrodes

- Indium Tin Oxide (ITO): ITO is one of the most widely used transparent conductive oxides due to its exceptional optical transparency and electrical conductivity [192,193,194]. This material achieves low sheet resistance and high transmittance, making it ideal for applications such as ECDs, touchscreens, and displays. ITO’s ability to form thin, uniform films via techniques such as sputtering and chemical vapor deposition (CVD) further enhances its versatility. However, ITO faces significant challenges, including brittleness, which drastically restricts its use in flexible devices, and the high cost of indium, which limits its widespread use for more low-end applications [195]. ITO remains a benchmark material for transparent conductive ceramics in rigid optoelectronic devices and is in that regard often compared to other materials.
- Fluorine-Doped Tin Oxide (FTO): FTO is among the most commonly used transparent conductive ceramics due to its high optical transparency and moderate electrical conductivity [193]. Its excellent stability under thermal and chemical stress makes it ideal for ECDs exposed to harsh environments. FTO is often used as a substrate for electrochromic coatings in smart windows and displays. However, its brittleness and high sheet resistance compared to materials like ITO limits its use in flexible applications.
- Antimony-Doped Tin Oxide (ATO): ATO is another tin oxide-based material offering excellent stability and durability under UV exposure [193]. While its conductivity is slightly lower than that of FTO, it exhibits superior thermal resistance [196]. However, high processing costs and limited flexibility present challenges for broader adoption.

#### 3.5.2. Zinc Oxide-Based Electrodes

- Aluminum-Doped Zinc Oxide (AZO): AZO is a low-cost electrical alternative to indium-based TCOs like ITO. It combines high transparency and conductivity with good environmental stability [194]. AZO’s flexibility when deposited on polymer substrates makes it suitable for flexible ECDs and wearable devices [197]. However, AZO is susceptible to degradation under high humidity or UV exposure, necessitating protective coatings to maintain long-term performance [198].
- Gallium-Doped Zinc Oxide (GZO): GZO provides comparable electrical conductivity and optical transparency to AZO but with enhanced chemical and thermal stability [199,200]. This makes GZO less prone to degradation, particularly in outdoor environments. As a result, it is frequently employed in applications such as smart windows and ECDs exposed to extreme conditions. Despite its advantages, GZO’s higher production cost compared to AZO can limit its widespread use.

#### 3.5.3. Indium-Free Transparent Conductive Ceramics

Indium-free TCCs are usually limited to use of doped titanium dioxide (TiO_2_) [201] or doped hafnium dioxide (HfO_2_) [202]. TiO_2_ doped with elements like niobium or tantalum is an emerging TCC that offers good conductivity and excellent environmental stability. Its high chemical resistance and optical transparency make it a promising candidate for TCEs in ECDs and photovoltaic systems. However, its conductivity is generally lower than that of tin and zinc oxides, which restricts its application in high-performance devices. Doped HfO_2_ is a much lesser-used experimental material that features high transparency and chemical stability that is often used in combination with other materials [203,204]. While still in early development stages, it holds some potential for next-generation TCEs. The high cost of production and limited availability are significant challenges to its commercialization and as such holds limited advantages to more established indium-doped alternatives.

TCCs in general exhibit high transparency in the visible spectrum, making them essential for optoelectronic applications such as displays and smart windows. Compared to conductive polymers, these materials are known for their good chemical, thermal, and UV stability, ensuring long-term performance even in harsh environmental conditions. However, sheet resistance, along with transmittance, varies substantially depending on the thickness of prepared TCCs. While FTO has excellent conductivity (4.5–20 Ω/sq), it is not often the first choice for flexible applications due to its brittleness [205]. ITO on the other hand is more eager to attach to flexible substrates and is thus more preferred, despite its lower sheet resistance (200–400 Ω/sq) [206]. Zinc oxide thin films, such as IZO, needs to stay fairly thin (6–12 nm) to remain flexible; however, thinner films often show higher sheet resistance (above 20 Ω/sq below 10 nm) which limits their use for large-scale applications such as smart windows [207]. In addition, the inherent brittleness of ceramics highly limits their application in flexible and wearable electronics [208]. Hybrid systems integrating ceramics with polymers or nanostructures are being developed to address this limitation [209,210]. As such, they are rarely employed in ECD applications, which require exposure to mechanical stress, as more flexible alternatives are often preferred. Typical TCCs are presented in the Table 6.

**Figure 5:**
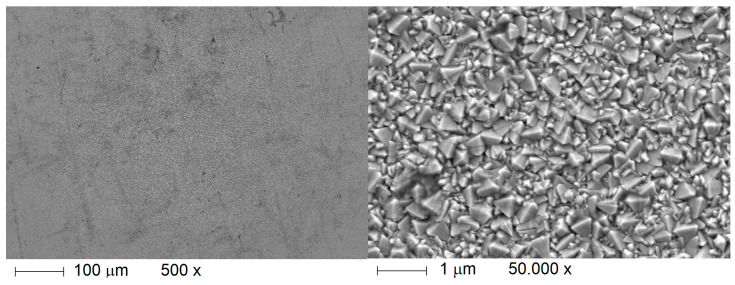
SEM image of an examples of commercial FTO thin film that is in use for electrochromic devices, solar cells, and other electronics.

A scanning electron microscope image of commercial FTO thin film prepared via sol-gel method is presented in Figure 5. It can be observed that prepared FTO is not of monolithic structure, but is in a polycrystalline form, neatly packed together.

#### 3.5.4. Manufacturing Methods of TCCs

Transparent conductive ceramics (TCCs) materials are typically processed into thin films using various deposition techniques, each tailored to achieve desired performance characteristics such as uniformity, electrical conductivity, and optical transmittance.

- Indium Tin Oxide (ITO)

The preparation of ITO thin films primarily involves physical and chemical vapor deposition techniques to ensure uniform and high-performance coatings:

  ○ Sputtering:

This is the most commonly employed method for ITO film deposition. In this process, an ITO target (90% In_2_O_3_ and 10% SnO_2_) is bombarded with high-energy ions in a vacuum chamber [211]. The ejected material is deposited as a thin film on a substrate, such as glass or flexible polymers. Sputtering allows precise control over film thickness and uniformity, and is compatible with large-area deposition, making it suitable for industrial applications. However, the process requires expensive vacuum systems and has relatively slow deposition rates.

  ○ Pulsed Laser Deposition (PLD):

A high-energy laser beam is directed onto an ITO target, vaporizing the material and depositing it on the substrate [218]. PLD achieves high-quality films with excellent adhesion but is generally limited to small-scale applications due to its low throughput and high cost.

  ○ Chemical or Physical Vapor Deposition (CVD or PVD):

ITO precursors are vaporized and reacted in a chamber to form thin films on heated substrates [212]. While the CVD/PVD process enables high deposition rates and good film quality, it often shows difficulties in maintaining film uniformity.

- Fluorine-Doped Tin Oxide (FTO)

FTO thin films are prepared from tin oxide (SnO_2_) doped with small amounts of fluorine. These films are typically deposited using techniques that ensure high stability and uniformity under thermal and chemical stress:

  ○ Spray Pyrolysis:

A solution containing tin precursors (e.g., SnCl_4_) and a fluorine source (e.g., NH_4_F) is sprayed onto a heated substrate [213]. The heat decomposes the precursors, forming a thin FTO film. Spray pyrolysis is a cost-effective method suitable for large-scale production, but it may result in uneven thickness and rough surface morphology.

  ○ Chemical Vapor Deposition (CVD):

Precursors like tin tetrachloride (SnCl_4_) and a fluorine dopant are reacted in a vapor phase on a heated substrate [214]. CVD produces high-quality FTO films with excellent uniformity and adhesion, but it requires precise control over reaction conditions and high initial investment.

  ○ Sputtering:

FTO films can also be deposited using sputtering, similar to ITO [219]. This method is particularly suitable for applications requiring precise thickness control and high film density.

- Aluminum-Doped Zinc Oxide (AZO)

AZO is a promising alternative to ITO, offering comparable properties at a significantly lower cost. The preparation of AZO films involves doping zinc oxide (ZnO) with aluminum, typically using physical and chemical deposition methods:

  ○ Sputtering:

A ZnO target doped with aluminum (1–5%) is used in a sputtering system to deposit AZO films [220]. This method ensures uniform coatings with precise control over film properties.

  ○ Chemical Vapor Deposition (CVD):

In CVD, gaseous precursors such as diethylzinc (DEZ) and trimethylaluminum (TMA) react to form AZO films on heated substrates [221]. This method provides excellent film quality but requires high temperatures, which may limit compatibility with flexible substrates.

  ○ Hydrothermal synthesis:

This method enables to prepare AZO from Al and Zn salt precursors in an autoclave at temperatures between 100 and 200 °C [222]. It provides a way to produce nanoparticles that can be later deposited with other solution process techniques.

  ○ Solution Processing:

Techniques like spin coating, dip coating, or spray coating are employed using a solution of ZnO and aluminum precursors [223,224]. Solution-based methods are cost-effective and scalable, but the resulting films often require post-deposition annealing to achieve optimal conductivity and transparency.

Prime materials for use in flexible applications are ITO and FTO; ITO films exhibit low sheet resistance and high optical transmittance, while requiring low temperature for coating preparation, making them an ideal choice. However, the reliance on indium raises concerns about scalability and long-term sustainability. AZO films are valued for their low cost, good environmental stability, and compatibility with flexible substrates, making them ideal for emerging applications such as wearable electronics and flexible displays. Overall, AZO has lower conductivity compared to ITO and is susceptible to degradation under high humidity or UV exposure, necessitating protective coatings or hybrid structures. While sputtering remains a dominant technique for achieving high-quality TCC films, alternative methods like spray pyrolysis [225] and solution processing [194] offer cost-effective routes for large-scale production.

## 4. Discussion

The field of electrochromic devices (ECDs) has reached a critical juncture, where the demand for advanced, scalable, and sustainable materials is driving innovation. Despite significant progress in electrode materials, challenges remain in optimizing their performance [26]. Scalability remains a significant challenge for materials like Ag nanowires and metal meshes, which perform exceptionally well in small-scale applications but encounter difficulties when adapted to large-area devices [182]. Manufacturing techniques such as roll-to-roll processing show promise but require further refinement to achieve consistent uniformity and cost-effectiveness [105]. Stability under extreme conditions is another critical concern, as electrodes must endure high humidity, extreme temperatures, and UV exposure. Materials like conductive polymers and nanocellulose composites require improved protective coatings to enhance their longevity and reliability under these demanding environmental conditions. Cost constraints also pose a barrier to the widespread adoption of high-performance materials such as CNTs and Ag nanowires, which are often cost-prohibitive for commercial-scale use. Developing low-cost alternatives or hybrid materials that balance performance and affordability is a key focus for researchers. Additionally, the integration of electrodes into flexible and wearable devices presents unique mechanical challenges, as these applications demand durability under repeated bending, stretching, and twisting. Advances in composite materials and substrate engineering are essential to overcome these obstacles and enable reliable performance in flexible and wearable technologies [39,226]. Opportunities in the development of ECD electrodes highlight exciting directions for future advancements. One promising avenue is the exploration of hybrid material systems [193], where different electrode materials, such as nanocellulose combined with CNTs or Ag nanowires, are integrated to achieve an optimal balance of conductivity, transparency, and flexibility. These hybrid systems leverage the strengths of each component while addressing their individual weaknesses. Another opportunity lies in biodegradable and sustainable materials, driven by the global push for environmentally friendly electronics [227,228]. Materials like nanocellulose and conductive polymers not only help reduce electronic waste but also align with sustainability goals, reducing the use of scarce materials like indium in the process. Advances in coating techniques also hold significant potential; innovations such as atomic layer deposition (ALD) [229] and molecular-level encapsulation [230] are being explored to enhance the chemical stability and durability of electrode materials, particularly under harsh environmental conditions. Additionally, functional integration offers an opportunity, as researchers aim to develop multifunctional electrodes that combine electrochromic properties with energy storage or sensing capabilities [32,231,232,233]. These integrated solutions expand the range of applications for ECDs without compromising key attributes like transparency and flexibility, opening up new possibilities for next-generation devices.

## 5. Conclusions

The future of electrochromic devices hinges on the continued advancement of electrode materials that are scalable, durable, and environmentally sustainable. By addressing current challenges and leveraging emerging opportunities, the field can unlock new applications and achieve widespread adoption. Hybrid systems and innovative manufacturing techniques represent key avenues for progress, ensuring that ECDs remain at the forefront of smart and sustainable technologies in the 21st century.

## Figures and Tables

**Table 1:** Transmittance and sheet resistance of AgNWs, with preparation methods.

Transmittance [%]	Sheet Resistance [Ω/sq]	Method	Scientific Paper
90	15.6	Polyol process	[22]
92.5	45	Polyol process	[63]
91.3	8.6	Polyol process	[64]
90	50	Spray deposit	[65]
80	8	Spin deposit	[60]
91	6.5	Electron beam lithography	[66]

**Table 2:** Transmittance and sheet resistance of metal meshes, with specific metal used.

Transmittance [%]	Sheet Resistance [Ω/sq]	Material	Scientific Paper
85.5	0.18	Cu	[94]
85	0.83	Cu	[95]
87.3	2.05	Steel (type 316)	[93]
65–89	16.5–104.5	Au	[92]
72	11	Ag	[96]

**Table 3:** Transmittance and sheet resistance of conductive polymers.

Transmittance [%]	Sheet Resistance [Ω/sq]	Polymer Type	Scientific Paper
81.1	1.5	PEDOT/PSS (on copper mesh)	[142]
90.4	32	PEDOT/PSS (HClO_4_ treatment)	[141]
90	84	PANI (with graphene)	[144]
70	1100	PANI	[143]
30	200	PPy	[145]
90	400–800	P3HT (with CNT)	[146]

**Table 4:** Key manufacturing methods for TCP and their advantages.

Polymer	Key Methods	Advantages
PEDOT/PSS	Solution processing, surface coating, electropolymerization	High scalability, compatibility with flexible substrates, enhanced electrical conductivity with post-treatments. Most commonly used.
PANI/PPy	Chemical oxidative polymerization, electropolymerization, blend/composite fabrication, spray/spin coating/composite fabrication	Tunable properties, cost-effective, adaptable for composites.
PT	Chemical oxidative polymerization, electrochemical polymerization, solution processing	Mechanical flexibility and environmentally stable.

**Table 5:** Transmittance and sheet resistance of CNTs.

Transmittance [%]	Sheet Resistance [Ω/sq]	Method	Scientific Paper
82.7	423	CVD	[173]
90	31	CVD (Au doped via drop casting)	[174]
90 (in IR area)	200	CVD	[175]

**Table 6:** Transmittance and sheet resistance of different TCC materials and their preferable method of manufacture.

Transmittance [%]	Sheet Resistance [Ω/sq]	Material	Method	Scientific Paper
75–90	200–400	ITO	Magnetron sputtering	[211]
87.4	39.6	ITO	PVD	[212]
86.2	42.7	IZO	PVD	[212]
75–85	10–20	FTO	Spray pyrolysis	[213]
80–84	4.5–8	FTO	Atm. Pressure CVD	[214]
80–85	25–40	AZO	RF sputtering	[215]
60–80	6 × 10^3^–1 × 10^4^	ATO	Spin coating	[216]
25–80%	2.5 × 10^5^–1 × 10^6^	HfO_2_	PLD	[217]

## Data Availability

No new data have been created during preparation of this review paper.

## References

[1] Mortimer R.J. (2011). Electrochromic materials. Annu. Rev. Mater. Res..

[2] Hamada H., Yano K., Take H., Inami Y., Matsuura M., Wada T. (1983). Electrochromic displays: Status and future prospects. Displays.

[3] Zhang X., Chang D., Liu J., Luo Y. (2010). Conducting polymer aerogels from supercritical CO_2_ drying PEDOT-PSS hydrogels. J. Mater. Chem..

[4] Kao S.-Y., Lu H.-C., Kung C.-W., Chen H.-W., Chang T.-H., Ho K.-C. (2016). Thermally cured dual functional viologen-based all-in-one electrochromic devices with panchromatic modulation. ACS Appl. Mater. Interfaces.

[5] Ohsuku T., Hirai T. (1982). An electrochromic display based on titanium dioxide. Electrochim. Acta.

[6] Schmidt D.J., Pridgen E.M., Hammond P.T., Love J.C. (2010). Layer-by-layer assembly of a ph-responsive and electrochromic thin film. J. Chem. Educ..

[7] Szmacinski H., Lakowicz J.R. (1995). Fluorescence lifetime-based sensing and imaging. Sens. Actuators B Chem..

[8] Dalisa A.L. (1977). Electrophoretic display technology. IEEE Trans. Electron Devices.

[9] Kulesza P.J., Malik M.A., Zamponi S., Berrettoni M., Marassi R. (1995). Electrolyte-cation-dependent coloring, electrochromism and thermochromism of cobalt(ii) hexacyanoferrate(iii, ii) films. J. Electroanal. Chem..

[10] Wang C., Li J., Zhang L., Fu S. (2022). Preparation and property optimization of bistable electrochromic microcapsules. Dye. Pigment..

[11] Sun X.W., Wang J.X. (2008). Fast switching electrochromic display using a viologen-modified ZnO nanowire array electrode. Nano Lett..

[12] Granqvist C.-G. (2013). Electrochromic metal oxides: An introduction to materials and devices. Electrochromic Materials and Devices.

[13] Wen H., Weng B., Wang B., Xiao W., Liu X., Wang Y., Zhang M., Huang H. (2024). Advancements in transparent conductive oxides for photoelectrochemical applications. Nanomaterials.

[14] Harris K.D., Elias A.L., Chung H.J. (2016). Flexible electronics under strain: A review of mechanical characterization and durability enhancement strategies. J. Mater. Sci..

[15] Huang S., Liu Y., Zhao Y., Ren Z., Guo C.F. (2019). Flexible electronics: Stretchable electrodes and their future. Adv. Funct. Mater..

[16] Huang L., Chen X., Wu X., Hu Z., Nie S., Huang C., Zhang S., Xu W., Pei F., Su W. (2023). Hybrid Ag/Ni mesh/PH 1000 transparent electrodes for high performance flexible electrochromic devices with exceptional stability. Flex. Print. Electron..

[17] Qiu T., Luo B., Liang M., Ning J., Wang B., Li X., Zhi L. (2015). Hydrogen reduced graphene oxide/metal grid hybrid film: Towards high performance transparent conductive electrode for flexible electrochromic devices. Carbon.

[18] Jung J., Cho H., Yuksel R., Kim D., Lee H., Kwon J., Lee P., Yeo J., Hong S., Unalan H.E. (2019). Stretchable/flexible silver nanowire electrodes for energy device applications. Nanoscale.

[19] Chen J., Minett A.I., Liu Y., Lynam C., Sherrell P., Wang C., Wallace G.G. (2008). Direct growth of flexible carbon nanotube electrodes. Adv. Mater..

[20] Tan R.K.L., Reeves S.P., Hashemi N., Thomas D.G., Kavak E., Montazami R., Hashemi N.N. (2017). Graphene as a flexible electrode: Review of fabrication approaches. J. Mater. Chem. A.

[21] Das T.K., Prusty S. (2012). Review on conducting polymers and their applications. Polym. Plast. Technol. Eng..

[22] Wang J., Jiu J., Araki T., Nogi M., Sugahara T., Nagao S., Koga H., He P., Suganuma K. (2015). Silver nanowire electrodes: Conductivity improvement without post-treatment and application in capacitive pressure sensors. Nano-Micro Lett..

[23] Spechler J.A., Koh T.-W., Herb J.T., Rand B.P., Arnold C.B. (2015). A transparent, smooth, thermally robust, conductive polyimide for flexible electronics. Adv. Funct. Mater..

[24] Jensen J., Hösel M., Dyer A.L., Krebs F.C. (2015). Development and manufacture of polymer-based electrochromic devices. Adv. Funct. Mater..

[25] Steinberg M.D., Kassal P., Steinberg I.M. (2016). System architectures in wearable electrochemical sensors. Electroanalysis.

[26] Cai G., Wang J., Lee P.S. (2016). Next-generation multifunctional electrochromic devices. Acc. Chem. Res..

[27] Li L., Yu Z., Ye C., Song Y. (2024). Structural color boosted electrochromic devices: Strategies and applications. Adv. Funct. Mater..

[28] Deb S.K. (1969). A novel electrophotographic system. Appl. Opt..

[29] Chang I.F., Gilbert B.L., Sun T.I. (1975). Electrochemichromic systems for display applications. J. Electrochem. Soc..

[30] Deb S.K., Witzke H. The solid state electrochromic phenomenon and its applications to display devices. Proceedings of the 1975 International Electron Devices Meeting.

[31] Gustafsson J.C., Liedberg B., Inganäs O. (1994). In situ spectroscopic investigations of electrochromism and ion transport in a poly (3,4-ethylenedioxythiophene) electrode in a solid state electrochemical cell. Solid State Ion..

[32] Theodosiou K., Giannopoulos P., Georgakopoulos T., Stathatos E. (2020). Quasi-solid-state electrochromic cells with energy storage properties made with inkjet printing. Materials.

[33] Liu H.-S., Pan B.-C., Liou G.-S. (2017). Highly transparent agnw/pdms stretchable electrodes for elastomeric electrochromic devices. Nanoscale.

[34] Chen W.-H., Li F.-W., Liou G.-S. (2019). Novel stretchable ambipolar electrochromic devices based on highly transparent AgNW/PDMS hybrid electrodes. Adv. Opt. Mater..

[35] Koo J., Amoli V., Kim S.Y., Lee C., Kim J., Park S.-M., Kim J., Ahn J.M., Jung K.J., Kim D.H. (2020). Low-power, deformable, dynamic multicolor electrochromic skin. Nano Energy.

[36] Varghese Hansen R., Yang J., Zheng L. (2018). Flexible electrochromic materials based on CNT/PDA hybrids. Adv. Colloid Interface Sci..

[37] Shi G., Fan H., Wang W., Hou C., Zhang Q., Li Y., Xiao H., Dai G., Li K., Wang H. (2024). Carbon nanotube-grid infrared transparent electrodes for flexible electrochromic devices with visible to mid-infrared dual-band modulation. Mater. Today Chem..

[38] Cao X., Lau C., Liu Y., Wu F., Gui H., Liu Q., Ma Y., Wan H., Amer M.R., Zhou C. (2016). Fully screen-printed, large-area, and flexible active-matrix electrochromic displays using carbon nanotube thin-film transistors. ACS Nano.

[39] Li W., Bai T., Fu G., Zhang Q., Liu J., Wang H., Sun Y., Yan H. (2022). Progress and challenges in flexible electrochromic devices. Sol. Energy Mater. Sol. Cells.

[40] Nuroldayeva G., Balanay M.P. (2023). Flexing the spectrum: Advancements and prospects of flexible electrochromic materials. Polymers.

[41] Yu J., Wang S., Gao L., Qiao G., Lin M.-F., Wei C., Chen J., Li S. (2025). Recent advances in flexible multifunctional electrochromic devices. Nanoscale.

[42] Habboush S., Rojas S., Rodríguez N., Rivadeneyra A. (2024). The role of interdigitated electrodes in printed and flexible electronics. Sensors.

[43] Agrawal V., Ghosh T., Kumar R., Singla E., Agnihotri P.K. (2024). Design and fabrication of dual electrochromic device with broader color space. J. Mater. Sci. Mater. Electron..

[44] Liang Z., Yukikawa M., Nakamura K., Kobayashi N. (2018). A novel organic electrochromic device with hybrid capacitor architecture towards multicolour representation. Phys. Chem. Chem. Phys..

[45] Bian C., Wang J., Liu H., Yan Y., Zhang P., Yang W., Jia S., Guo X., Cai G. (2024). Complementary multicolor electrochromic devices with excellent stability based on porous tin oxide nanosheet scaffold. Nano Res..

[46] Shinde M.A., Ahmad K., Kim H. (2024). Fabrication of PEDOT:PSS/WO_3_ films on indium tin oxide based glass and flexible substrates for smart windows application. Opt. Mater..

[47] Gurudevi P., Venkateswari P., Sivakumar T., Ramesh C., Vanitha P. (2023). Solution processed WO_3_ and PEDOT:PSS composite for hole transport layer in ITO-free organic solar cells. J. Clust. Sci..

[48] Nie S., Ning C., Liu Y., Lian Y., Zhao L., Liu Z. (2024). Enhanced electrochromic performance of WO_3_/PEDOT by π-electron conjugation system. Ceram. Int..

[49] Lee J., Lee Y., Ahn J., Kim J., Yoon S., Kim Y.S., Cho K.Y. (2017). Improved electrochromic device performance from silver grid on flexible transparent conducting electrode prepared by electrohydrodynamic jet printing. J. Mater. Chem. C.

[50] Jeong S.-J., Jo M.-H., Ahn H.-J. (2023). 3D-printed film architecture via automatic micro 3D-printing system: Micro-intersection engineering of V_2_O_5_ thin/thick films for ultrafast electrochromic energy storage devices. Chem. Eng. J..

[51] Marques A., Santos L., Pereira S., Emanuele U., Sinopoli S., Igreja R., Sales G., Martins R., Fortunato E. (2018). A planar electrochromic device using WO_3_ nanoparticles and a modified paper-based electrolyte. Proceedings.

[52] Barquinha P., Pereira S., Pereira L., Wojcik P., Grey P., Martins R., Fortunato E. (2015). Flexible and transparent WO_3_ transistor with electrical and optical modulation. Adv. Electron. Mater..

[53] Grey P., Pereira L., Pereira S., Barquinha P., Cunha I., Martins R., Fortunato E. (2016). Transistors: Solid state electrochemical WO_3_ transistors with high current modulation. Adv. Electron. Mater..

[54] Deb S.K. (2008). Opportunities and challenges in science and technology of WO_3_ for electrochromic and related applications. Sol. Energy Mater. Sol. Cells.

[55] Monk P., Mortimer R., Rosseinsky D. (2007). A brief history of electrochromism. Electrochromism and Electrochromic Devices.

[56] Rozman M., Gaberšček M., Marolt G., Bren U., Lukšič M. (2019). An inverted sandwich electrochromic device architecture does not require optically transparent electrodes. Adv. Mater. Technol..

[57] Zheng J.Y., Sun Q., Cui J., Yu X., Li S., Zhang L., Jiang S., Ma W., Ma R. (2023). Review on recent progress in WO_3_-based electrochromic films: Preparation methods and performance enhancement strategies. Nanoscale.

[58] Rozman M., Žener B., Matoh L., Godec R.F., Mourtzikou A., Stathatos E., Bren U., Lukšič M. (2020). Flexible electrochromic tape using steel foil with WO_3_ thin film. Electrochim. Acta.

[59] Lu H.-Y., Chou C.-Y., Wu J.-H., Lin J.-J., Liou G.-S. (2015). Highly transparent and flexible polyimide–AgNW hybrid electrodes with excellent thermal stability for electrochromic applications and defogging devices. J. Mater. Chem. C.

[60] Shinde M.A., Kim H. (2021). Highly stable silver nanowire-based transparent conductive electrodes for electrochromic devices. Mater. Today Commun..

[61] Fisk Z., Webb G.W., Fradin F.Y. (1981). 5—Electrical resistivity of metals. Treatise on Materials Science & Technology.

[62] Lee J.-Y., Connor S.T., Cui Y., Peumans P. (2008). Solution-processed metal nanowire mesh transparent electrodes. Nano Lett..

[63] Zhang W., Chen X., Zhang G., Wang S., Zhu S., Wu X., Wang Y., Wang Q., Hu C. (2019). Conducting polymer/silver nanowires stacking composite films for high-performance electrochromic devices. Sol. Energy Mater. Sol. Cells.

[64] Xu F., Xu W., Mao B., Shen W., Yu Y., Tan R., Song W. (2018). Preparation and cold welding of silver nanowire based transparent electrodes with optical transmittances >90% and sheet resistances < 10 ohm/sq. J. Colloid Interface Sci..

[65] Scardaci V., Coull R., Lyons P.E., Rickard D., Coleman J.N. (2011). Spray deposition of highly transparent, low-resistance networks of silver nanowires over large areas. Small.

[66] van de Groep J., Spinelli P., Polman A. (2012). Transparent conducting silver nanowire networks. Nano Lett..

[67] Hemmati S., Harris M.T., Barkey D.P. (2020). Polyol silver nanowire synthesis and the outlook for a green process. J. Nanomater..

[68] Kim T., Canlier A., Kim G.H., Choi J., Park M., Han S.M. (2013). Electrostatic spray deposition of highly transparent silver nanowire electrode on flexible substrate. ACS Appl. Mater. Interfaces.

[69] Teymouri Z., Naji L., Fakharan Z. (2018). The influences of polyol process parameters on the optoelectronic characteristics of AgNWs-based flexible electrodes and their application in ITO-free polymer solar cells. Org. Electron..

[70] Li J., Tao Y., Chen S., Li H., Chen P., Wei M.-Z., Wang H., Li K., Mazzeo M., Duan Y. (2017). A flexible plasma-treated silver-nanowire electrode for organic light-emitting devices. Sci. Rep..

[71] Kim S., Kim S.Y., Kim J., Kim J.H. (2014). Highly reliable AGNW/PEDOT:PSS hybrid films: Efficient methods for enhancing transparency and lowering resistance and haziness. J. Mater. Chem. C.

[72] Liu Y.-S., Feng J., Ou X.-L., Cui H.-F., Xu M., Sun H.-B. (2016). Ultrasmooth, highly conductive and transparent PEDOT:PSS/silver nanowire composite electrode for flexible organic light-emitting devices. Org. Electron..

[73] Ahn Y., Jeong Y., Lee Y. (2012). Improved thermal oxidation stability of solution-processable silver nanowire transparent electrode by reduced graphene oxide. ACS Appl. Mater. Interfaces.

[74] Guan F., He H., Li Q., Shen Y., Kang F., Zhang C., Zhai H. (2024). Transparent conductive films based on silver nanowires and SiO_2_ nanoparticles for flexible electronics. ACS Appl. Nano Mater..

[75] Ma Y., Sim G.W., Jo S., Hyun D.C., Roh J.-S., Ko D., Kim J. (2024). Stability of silver-nanowire-based flexible transparent electrodes under mechanical stress. Appl. Sci..

[76] Mayousse C., Celle C., Fraczkiewicz A., Simonato J.-P. (2015). Stability of silver nanowire based electrodes under environmental and electrical stresses. Nanoscale.

[77] Yun T.G., Hwang B. (2021). Effect of mechanical properties of substrates on flexibility of Ag nanowire electrodes under a large number of bending cycles. Coatings.

[78] Hao T., Wang S., Xu H., Zhang X., Xue J., Liu S., Song Y., Li Y., Zhao J. (2021). Highly robust, transparent, and conductive films based on AgNW-c nanowires for flexible smart windows. Appl. Surf. Sci..

[79] Ding Y., Cui Y., Liu X., Liu G., Shan F. (2020). Welded silver nanowire networks as high-performance transparent conductive electrodes: Welding techniques and device applications. Appl. Mater. Today.

[80] Lee H.B., Jin W.-Y., Ovhal M.M., Kumar N., Kang J.-W. (2019). Flexible transparent conducting electrodes based on metal meshes for organic optoelectronic device applications: A review. J. Mater. Chem. C.

[81] Zhou H., Song Y. (2021). Fabrication of silver mesh/grid and its applications in electronics. ACS Appl. Mater. Interfaces.

[82] Kim W.-K., Lee S., Hee Lee D., Hee Park I., Seong Bae J., Woo Lee T., Kim J.-Y., Hun Park J., Chan Cho Y., Ryong Cho C. (2015). Cu mesh for flexible transparent conductive electrodes. Sci. Rep..

[83] Yavuz A., Kaplan K., Bedir M. (2021). Copper oxide coated stainless steel mesh for flexible electrodes. J. Phys. Chem. Solids.

[84] Kummer M., Kirchhoff J.R. (1993). Graphite-coated metal mesh optically transparent electrodes. Anal. Chem..

[85] Ali M.M., Hussain A., Song R.-H., Khan M.Z., Park S.-J., Ishfaq H.A., Joh D.W., Hong J.-E., Lee S.-B., Lim T.-H. (2024). Beyond traditional fuel cells: Development and a comprehensive analysis of mechanically robust metal mesh-supported solid oxide fuel cell. Ceram. Int..

[86] Ade P., Pisano G., Tucker C., Weaver S. (2006). A Review of Metal Mesh Filters.

[87] Megremis S.J., Baltzer N., Copponnex T. (2014). 3—Corrosion resistance of precious metals for biomedical applications. Precious Metals for Biomedical Applications.

[88] Jäger H., Grove E.L. (1978). Precious metals. Applied Atomic Spectroscopy.

[89] Zhang Z.L., Bell T. (1985). Structure and corrosion resistance of plasma nitrided stainless steel. Surf. Eng..

[90] Zhao S., Xie X., Smith G.D., Patel S.J. (2006). Research and improvement on structure stability and corrosion resistance of nickel-base superalloy INCONEL alloy 740. Mater. Des..

[91] Patil A.R., Vagge S.T. (2022). Hot corrosion behaviour of INCONEL 738 superalloy in presence of NaCl, Na_2_SO_4_, V_2_O_5_. Mater. Today Proc..

[92] Li Z., Wang G., Li Z., Cheng Z., Zhou G., Li S. (2019). Flexible transparent electrodes based on gold nanomeshes. Nanoscale Res. Lett..

[93] Yeang A.L., Hernandez T.S., Strand M.T., Slotcavage D.J., Abraham E., Smalyukh I.I., Barile C.J., McGehee M.D. (2022). Transparent, high-charge capacity metal mesh electrode for reversible metal electrodeposition dynamic windows with dark-state transmission < 0.1%. Adv. Energy Mater..

[94] Chen Z., Yang S., Huang J., Gu Y., Huang W., Liu S., Lin Z., Zeng Z., Hu Y., Chen Z. (2024). Flexible, transparent and conductive metal mesh films with ultra-high FoM for stretchable heating and electromagnetic interference shielding. Nano-Micro Lett..

[95] Walia S., Singh A.K., Rao V.S.G., Bose S., Kulkarni G.U. (2020). Metal mesh-based transparent electrodes as high-performance EMI shields. Bull. Mater. Sci..

[96] Witczak Ł., Chrzanowski M., Sitarek P., Łysień M., Podhorodecki A. (2023). Flexible quantum-dot light-emitting diodes using embedded silver mesh transparent electrodes manufactured by an ultraprecise deposition method. ACS Omega.

[97] Al-Qwairi F.O., Shaheen Shah S., Shabi A.H., Khan A., Aziz M.A. (2024). Stainless steel mesh in electrochemistry: Comprehensive applications and future prospects. Chem. Asian J..

[98] Khan A., Lee S., Jang T., Xiong Z., Zhang C., Tang J., Guo L.J., Li W.-D. (2016). High-performance flexible transparent electrode with an embedded metal mesh fabricated by cost-effective solution process. Small.

[99] Heilmeier G.H., Zanoni L.A., Barton L.A. (1968). Dynamic scattering: A new electrooptic effect in certain classes of nematic liquid crystals. Proc. IEEE.

[100] Jung H., Seo J.A., Choi S. (2017). Wearable atmospheric pressure plasma fabrics produced by knitting flexible wire electrodes for the decontamination of chemical warfare agents. Sci. Rep..

[101] Yang L., Iskander A., Mohammed M., and Jang B.Z. (2007). Nano-fabrication: A review. J. Chin. Inst. Eng..

[102] Barcelo S., Li Z. (2016). Nanoimprint lithography for nanodevice fabrication. Nano Converg..

[103] Huh J.W., Lee D.K., Jeon H.-J., Ahn C.W. (2016). New approach for fabricating hybrid-structured metal mesh films for flexible transparent electrodes by the combination of electrospinning and metal deposition. Nanotechnology.

[104] Meng X., Hu X., Yang X., Yin J., Wang Q., Huang L., Yu Z., Hu T., Tan L., Zhou W. (2018). Roll-to-roll printing of meter-scale composite transparent electrodes with optimized mechanical and optical properties for photoelectronics. ACS Appl. Mater. Interfaces.

[105] Hakola L., Jansson E., Futsch R., Happonen T., Thenot V., Depres G., Rougier A., Smolander M. (2021). Sustainable roll-to-roll manufactured multi-layer smart label. Int. J. Adv. Manuf. Technol..

[106] Seong B., Yoo H., Nguyen V.D., Jang Y., Ryu C., Byun D. (2014). Metal-mesh based transparent electrode on a 3-d curved surface by electrohydrodynamic jet printing. J. Micromech. Microeng..

[107] Choi Y.-M., Lee E.-S., Lee T.-M., Kim K.-Y. (2015). Optimization of a reverse-offset printing process and its application to a metal mesh touch screen sensor. Microelectron. Eng..

[108] Layani M., Darmawan P., Foo W.L., Liu L., Kamyshny A., Mandler D., Magdassi S., Lee P.S. (2014). Nanostructured electrochromic films by inkjet printing on large area and flexible transparent silver electrodes. Nanoscale.

[109] Qi X., Zhou J., Zhu X., Li H., Zhang G., Sun L., Wang R., Huang Y., Yang W., Zhang Y.-F. (2023). Microscale hybrid 3D printed ultrahigh aspect ratio embedded silver mesh for flexible transparent electrodes. Mater. Today Phys..

[110] Zhang Y., Wang X., Wang C., Liu J., Zhai H., Liu B., Zhao X., Fang D. (2018). Facile fabrication of zinc oxide coated superhydrophobic and superoleophilic meshes for efficient oil/water separation. RSC Adv..

[111] Xu S., Wang Q., Wang N., Zheng X. (2020). Fabrication of hierarchical structures on steel mesh with good superhydrophobicity and anti-corrosion property. Colloids Surf. A Physicochem. Eng. Asp..

[112] del Amo B., Véleva L., Di Sarli A.R., Elsner C.I. (2004). Performance of coated steel systems exposed to different media: Part i. Painted galvanized steel. Prog. Org. Coat..

[113] Zhang H., Tian Y., Wang S., Huang Y., Wen J., Hang C., Zheng Z., Wang C. (2020). Highly stable flexible transparent electrode via rapid electrodeposition coating of Ag-Au alloy on copper nanowires for bifunctional electrochromic and supercapacitor device. Chem. Eng. J..

[114] Zhao Z.-J., Ko J., Ahn J., Bok M., Gao M., Hwang S.H., Kang H.-J., Jeon S., Park I., Jeong J.-H. (2020). 3D layer-by-layer pd-containing nanocomposite platforms for enhancing the performance of hydrogen sensors. ACS Sens..

[115] Liu P., He G., Wu L. (2009). Structure deformation and failure of sintered steel wire mesh under torsion loading. Mater. Des..

[116] Zhu X., Liu M., Qi X., Li H., Zhang Y.-F., Li Z., Peng Z., Yang J., Qian L., Xu Q. (2021). Templateless, plating-free fabrication of flexible transparent electrodes with embedded silver mesh by electric-field-driven microscale 3D printing and hybrid hot embossing. Adv. Mater..

[117] Chen X., Guo W., Xie L., Wei C., Zhuang J., Su W., Cui Z. (2017). Embedded Ag/Ni metal-mesh with low surface roughness as transparent conductive electrode for optoelectronic applications. ACS Appl. Mater. Interfaces.

[118] Wang Y.-Y., Li B.-J., Huang L.-J., Xu Q. (2022). Fabrication and performances of silver grid transparent conducting films and heaters on glass and pet substrates based on fractal periodic grid pattern design. Surf. Interfaces.

[119] Nie B., Wang C., Li X., Tian H., Chen X., Liu G., Qiu Y., Shao J. (2021). High-performance transparent and conductive films with fully enclosed metal mesh. ACS Appl. Mater. Interfaces.

[120] Zhang P., Sui Q., Liu Z., Hu C., Li C., Guo X., Wang J., Cai G. (2024). Highly stable Ag@Au nanowires micromesh as transparent conductive network for stretchable electrochromic modulator. Chem. Eng. J..

[121] Kumar D., Sharma R.C. (1998). Advances in conductive polymers. Eur. Polym. J..

[122] Wolfart F., Hryniewicz B.M., Góes M.S., Corrêa C.M., Torresi R., Minadeo M.A.O.S., Córdoba de Torresi S.I., Oliveira R.D., Marchesi L.F., Vidotti M. (2017). Conducting polymers revisited: Applications in energy, electrochromism and molecular recognition. J. Solid State Electrochem..

[123] Mortimer R.J., Dyer A.L., Reynolds J.R. (2006). Electrochromic organic and polymeric materials for display applications. Displays.

[124] Sun K., Zhang S., Li P., Xia Y., Zhang X., Du D., Isikgor F.H., Ouyang J. (2015). Review on application of PEDOTS and PEDOT:PSS in energy conversion and storage devices. J. Mater. Sci. Mater. Electron..

[125] Kim J., Jang J.G., Hong J.-I., Kim S.H., Kwak J. (2016). Sulfuric acid vapor treatment for enhancing the thermoelectric properties of PEDOT:PSS thin-films. J. Mater. Sci. Mater. Electron..

[126] Lingstedt L.V., Ghittorelli M., Lu H., Koutsouras D.A., Marszalek T., Torricelli F., Crăciun N.I., Gkoupidenis P., Blom P.W.M. (2019). Effect of DMSO solvent treatments on the performance of PEDOT:PSS based organic electrochemical transistors. Adv. Electron. Mater..

[127] Okuzaki H., Harashina Y., Yan H. (2009). Highly conductive PEDOT/PSS microfibers fabricated by wet-spinning and dip-treatment in ethylene glycol. Eur. Polym. J..

[128] Zhao L., Zhao L., Xu Y., Qiu T., Zhi L., Shi G. (2009). Polyaniline electrochromic devices with transparent graphene electrodes. Electrochim. Acta.

[129] Lacroix J.C., Kanazawa K.K., Diaz A. (1989). Polyaniline: A very fast electrochromic material. J. Electrochem. Soc..

[130] Camurlu P. (2014). Polypyrrole derivatives for electrochromic applications. RSC Adv..

[131] Ratautaite V., Bagdziunas G., Ramanavicius A., Ramanaviciene A. (2019). An application of conducting polymer polypyrrole for the design of electrochromic PH and CO_2_ sensors. J. Electrochem. Soc..

[132] Girotto E.M., de Paoli M.-A. (1998). Polypyrrole color modulation and electrochromic contrast enhancement by doping with a dye. Adv. Mater..

[133] Miah M.R., Yang M., Khandaker S., Bashar M.M., Alsukaibi A.K.D., Hassan H.M.A., Znad H., Awual M.R. (2022). Polypyrrole-based sensors for volatile organic compounds (VOCs) sensing and capturing: A comprehensive review. Sens. Actuators A Phys..

[134] Wang J., Liu J., Hu M., Zeng J., Mu Y., Guo Y., Yu J., Ma X., Qiu Y., Huang Y. (2018). A flexible, electrochromic, rechargeable Zn//PPy battery with a short circuit chromatic warning function. J. Mater. Chem. A.

[135] Medrano-Solís A., Nicho M.E., Hernández-Guzmán F. (2017). Study of dual electrochromic devices based on polyaniline and poly (3-hexylthiophene) thin films. J. Mater. Sci. Mater. Electron..

[136] Baray-Calderón A., Camacho-Cáceres J., Hernández-Guzmán F., Hu H., Nicho M.E. (2023). Enhanced performance of poly(3-hexylthiophene)-based electrochromic devices by adding a mesoporous TiO_2_ layer. Synth. Met..

[137] Shin H., Kim Y., Bhuvana T., Lee J., Yang X., Park C., Kim E. (2012). Color combination of conductive polymers for black electrochromism. ACS Appl. Mater. Interfaces.

[138] Bezgin Carbas B. (2022). Fluorene based electrochromic conjugated polymers: A review. Polymer.

[139] Shin D.H., Choi S.-H. (2018). Recent studies of semitransparent solar cells. Coatings.

[140] Carter J.L., Kelly C.A., Marshall J.E., Jenkins M.J. (2024). Effect of thickness on the electrical properties of PEDOT:PSS/tween 80 films. Polym. J..

[141] Fan X., Stott N.E., Zeng J., Li Y., Ouyang J., Chu L., Song W. (2023). PEDOT:PSS materials for optoelectronics, thermoelectrics, and flexible and stretchable electronics. J. Mater. Chem. A.

[142] Yan C., Zhao L., Yu S. (2024). High-performance PEDOT: PSS/Cu mesh flexible transparent conductors with enhanced durability, adhesion and stability. J. Mater. Sci. Mater. Electron..

[143] Kim B.R., Lee H.K., Kim E., Lee S.-H. (2010). Intrinsic electromagnetic radiation shielding/absorbing characteristics of polyaniline-coated transparent thin films. Synth. Met..

[144] Sharma A.K., Sharma A.K., Sharma R., Sharma P., Singh S. (2024). Multifunctional transparent conductive flexible sensor based on graphene/polyaniline/graphene sandwich composite on PDMS substrate. IEEE Sens. J..

[145] Qi G., Wu Z., Wang H. (2013). Highly conductive and semitransparent free-standing polypyrrole films prepared by chemical interfacial polymerization. J. Mater. Chem. C.

[146] Lee S., Yeo J.-S., Ji Y., Cho C., Kim D.-Y., Na S.-I., Lee B.H., Lee T. (2012). Flexible organic solar cells composed of P3HT:Pcbm using chemically doped graphene electrodes. Nanotechnology.

[147] Kim N., Kee S., Lee S.H., Lee B.H., Kahng Y.H., Jo Y.-R., Kim B.-J., Lee K. (2014). Highly conductive PEDOT:PSS nanofibrils induced by solution-processed crystallization. Adv. Mater..

[148] Andrei V., Bethke K., Madzharova F., Beeg S., Knop-Gericke A., Kneipp J., Rademann K. (2017). Size dependence of electrical conductivity and thermoelectric enhancements in spin-coated PEDOT:PSS single and multiple layers. Adv. Electron. Mater..

[149] Gong F., Meng C., He J., Dong X. (2018). Fabrication of highly conductive and multifunctional polyester fabrics by spray-coating with PEDOT:PSS solutions. Prog. Org. Coat..

[150] Yamamoto S., Miyako R., Maeda R., Ishizaki Y., Mitsuishi M. (2023). Dip coating of water-resistant PEDOT:PSS films based on physical crosslinking. Macromol. Mater. Eng..

[151] Buga C., Viana J.C. (2022). Optimization of print quality of inkjet printed PEDOT:PSS patterns. Flex. Print. Electron..

[152] Benoudjit A., Bader M.M., Wan Salim W.W.A. (2018). Study of electropolymerized PEDOT:PSS transducers for application as electrochemical sensors in aqueous media. Sens. Bio-Sens. Res..

[153] Abu-Thabit N.Y. (2016). Chemical oxidative polymerization of polyaniline: A practical approach for preparation of smart conductive textiles. J. Chem. Educ..

[154] Kang H.C., Geckeler K.E. (2000). Enhanced electrical conductivity of polypyrrole prepared by chemical oxidative polymerization: Effect of the preparation technique and polymer additive. Polymer.

[155] Díez-Pascual A.M. (2020). Carbon-based polymer nanocomposites for high-performance applications. Polymers.

[156] Abaci U., Guney H.Y., Kadiroglu U. (2013). Morphological and electrochemical properties of PPy, PAni bilayer films and enhanced stability of their electrochromic devices (PPy/PAni–PEDOT, PAni/PPy–PEDOT). Electrochim. Acta.

[157] Sonavane A.C., Inamdar A.I., Dalavi D.S., Deshmukh H.P., Patil P.S. (2010). Simple and rapid synthesis of nio/ppy thin films with improved electrochromic performance. Electrochim. Acta.

[158] Reddy B.N., Kumar P.N., Deepa M. (2015). A poly(3,4-ethylenedioxypyrrole)–Au@WO_3_-based electrochromic pseudocapacitor. ChemPhysChem.

[159] Rasmussen S.C., Pickens J.C., Hutchison J.E. (1998). A new, general approach to tuning the properties of functionalized polythiophenes:  The oxidative polymerization of monosubstituted bithiophenes. Chem. Mater..

[160] Dixon A.G., Visvanathan R., Clark N.A., Stingelin N., Kopidakis N., Shaheen S.E. (2018). Molecular weight dependence of carrier mobility and recombination rate in neat P3HT films. J. Polym. Sci. Part B Polym. Phys..

[161] Si P., Chi Q., Li Z., Ulstrup J., Møller P.J., Mortensen J. (2007). Functional polythiophene nanoparticles:  Size-controlled electropolymerization and ion selective response. J. Am. Chem. Soc..

[162] Xu Z., Horowitz G., Garnier F. (1988). Cathodic electropolymerization of polythiophene on platinum and various semiconducting electrodes. J. Electroanal. Chem. Interfacial Electrochem..

[163] Shibuya Y., Mori A. (2020). Dehalogenative or deprotonative? The preparation pathway to the organometallic monomer for transition-metal-catalyzed catalyst-transfer-type polymerization of thiophene derivatives. Chem. A Eur. J..

[164] Youm S.G., Hwang E., Chavez C.A., Li X., Chatterjee S., Lusker K.L., Lu L., Strzalka J., Ankner J.F., Losovyj Y. (2016). Polythiophene thin films by surface-initiated polymerization: Mechanistic and structural studies. Chem. Mater..

[165] Lai Y.-Y., Tung T.-C., Liang W.-W., Cheng Y.-J. (2015). Synthesis of poly(3-hexylthiophene), poly(3-hexylselenophene), and poly(3-hexylselenophene-alt-3-hexylthiophene) by direct c–h arylation polymerization via n-heterocyclic carbene palladium catalysts. Macromolecules.

[166] Lim E.L., Yap C.C., Mat Teridi M.A., Teh C.H., Mohd Yusoff A.R.B., Hj Jumali M.H. (2016). A review of recent plasmonic nanoparticles incorporated P3HT: PCBM organic thin film solar cells. Org. Electron..

[167] Sekine C., Tsubata Y., Yamada T., Kitano M., Doi S. (2014). Recent progress of high performance polymer OLED and OPV materials for organic printed electronics. Sci. Technol. Adv. Mater..

[168] Huq A.F., Ammar A., Al-Enizi A.M., Karim A. (2017). In-situ orientation and crystal growth kinetics of P3HT in drop cast P3HT:Pcbm films. Polymer.

[169] Liu H., Li Q., Zhang S., Yin R., Liu X., He Y., Dai K., Shan C., Guo J., Liu C. (2018). Electrically conductive polymer composites for smart flexible strain sensors: A critical review. J. Mater. Chem. C.

[170] Wen Y., Xu J. (2017). Scientific importance of water-processable PEDOT–PSS and preparation, challenge and new application in sensors of its film electrode: A review. J. Polym. Sci. Part A Polym. Chem..

[171] Chen Y., Lyu M., Zhang Z., Yang F., Li Y. (2022). Controlled preparation of single-walled carbon nanotubes as materials for electronics. ACS Cent. Sci..

[172] Kukovecz Á., Kozma G., Kónya Z., Vajtai R. (2013). Multi-walled carbon nanotubes. Springer Handbook of Nanomaterials.

[173] Tian Y., Zhang X., Geng H.-Z., Yang H.-J., Li C., Da S.-X., Lu X., Wang J., Jia S.-L. (2017). Carbon nanotube/polyurethane films with high transparency, low sheet resistance and strong adhesion for antistatic application. RSC Adv..

[174] Goldt A.E., Zaremba O.T., Bulavskiy M.O., Fedorov F.S., Larionov K.V., Tsapenko A.P., Popov Z.I., Sorokin P., Anisimov A.S., Inani H. (2021). Highly efficient bilateral doping of single-walled carbon nanotubes. J. Mater. Chem. C.

[175] Hu L., Hecht D.S., Grüner G. (2009). Infrared transparent carbon nanotube thin films. Appl. Phys. Lett..

[176] Pełech I. (2010). Preparation of carbon nanotubes using cvd CVD method. Pol. J. Chem. Technol..

[177] Kim H.H., Kim H.J. (2006). The preparation of carbon nanotubes by dc arc discharge using a carbon cathode coated with catalyst. Mater. Sci. Eng. B.

[178] Ismail R.A., Mohsin M.H., Ali A.K., Hassoon K.I., Erten-Ela S. (2020). Preparation and characterization of carbon nanotubes by pulsed laser ablation in water for optoelectronic application. Phys. E Low-Dimens. Syst. Nanostructures.

[179] Choi G.S., Cho Y.S., Son K.H., Kim D.J. (2003). Mass production of carbon nanotubes using spin-coating of nanoparticles. Microelectron. Eng..

[180] Tuukkanen S., Välimäki M., Lehtimäki S., Vuorinen T., Lupo D. (2016). Behaviour of one-step spray-coated carbon nanotube supercapacitor in ambient light harvester circuit with printed organic solar cell and electrochromic display. Sci. Rep..

[181] Vaisman L., Wagner H.D., Marom G. (2006). The role of surfactants in dispersion of carbon nanotubes. Adv. Colloid Interface Sci..

[182] Fu G., Gong H., Bai T., Zhang Q., Wang H. (2023). Progress and challenges in wearable electrochromic devices: A review. J. Mater. Sci. Mater. Electron..

[183] Shen K.-Y., Hu C.-W., Chang L.-C., Ho K.-C. (2012). A complementary electrochromic device based on carbon nanotubes/conducting polymers. Sol. Energy Mater. Sol. Cells.

[184] Hu F., Yan B., Sun G., Xu J., Gu Y., Lin S., Zhang S., Liu B., Chen S. (2019). Conductive polymer nanotubes for electrochromic applications. ACS Appl. Nano Mater..

[185] Zhao Z., Yang Z., Hu Y., Li J., Fan X. (2013). Multiple functionalization of multi-walled carbon nanotubes with carboxyl and amino groups. Appl. Surf. Sci..

[186] Mallakpour S., Soltanian S. (2016). Surface functionalization of carbon nanotubes: Fabrication and applications. RSC Adv..

[187] Kuzubasoglu B.A., Sayar E., Bahadir S.K. (2021). Inkjet-printed CNT/PEDOT:PSS temperature sensor on a textile substrate for wearable intelligent systems. IEEE Sens. J..

[188] Ellmer K. (2012). Past achievements and future challenges in the development of optically transparent electrodes. Nat. Photonics.

[189] Granqvist C.G. (2007). Transparent conductors as solar energy materials: A panoramic review. Sol. Energy Mater. Sol. Cells.

[190] Fortunato E., Ginley D., Hosono H., Paine D.C. (2011). Transparent conducting oxides for photovoltaics. MRS Bull..

[191] Granqvist C.G., Azens A., Heszler P., Kish L.B., Österlund L. (2007). Nanomaterials for benign indoor environments: Electrochromics for “smart windows”, sensors for air quality, and photo-catalysts for air cleaning. Sol. Energy Mater. Sol. Cells.

[192] Goldner R.B., Foley G., Goldner E.L., Norton P., Wong K., Haas T., Seward G., Chapman R. (1985). Electrochromic behavior in ITO and related oxides. Appl. Opt..

[193] Rai V., Singh R.S., Blackwood D.J., Zhili D. (2020). A review on recent advances in electrochromic devices: A material approach. Adv. Eng. Mater..

[194] Patel J., Sharme R.K., Quijada M.A., Rana M.M. (2024). A review of transparent conducting films (TCFs): Prospective ITO and AZO deposition methods and applications. Nanomaterials.

[195] Gu C., Jia A.-B., Zhang Y.-M., Zhang S.X.-A. (2022). Emerging electrochromic materials and devices for future displays. Chem. Rev..

[196] Montero J., Guillén C., Herrero J. (2012). Nanocrystalline antimony doped tin oxide (ATO) thin films: A thermal restructuring study. Surf. Coat. Technol..

[197] Aziz D.M., Hassan S.A., Aziz S.B. (2024). Synthesis and characterization of enhanced azo-azomethine doped PANI/HCL conducting polymers for electrochemical applications. Sci. Rep..

[198] Mirletz H.M., Peterson K.A., Martin I.T., French R.H. (2015). Degradation of transparent conductive oxides: Interfacial engineering and mechanistic insights. Sol. Energy Mater. Sol. Cells.

[199] Nishi Y., Kasai Y., Suzuki R., Matsubara M., Muramatsu A., Kanie K. (2020). Gallium-doped zinc oxide nanoparticle thin films as transparent electrode materials with high conductivity. ACS Appl. Nano Mater..

[200] Khan S., Stamate E. (2022). Comparative study of aluminum-doped zinc oxide, gallium-doped zinc oxide and indium-doped tin oxide thin films deposited by radio frequency magnetron sputtering. Nanomaterials.

[201] Berger T., Monllor-Satoca D., Jankulovska M., Lana-Villarreal T., Gómez R. (2012). The electrochemistry of nanostructured titanium dioxide electrodes. ChemPhysChem.

[202] Bilal U., Ramzan M., Imran M., Naz G., Mukhtar M.W., Fahim F., Iqbal H.M.N. (2022). HfO_2_-based nanostructured thin-films (i.e., low-e coatings) with robust optical performance and energy efficiency. J. Nanostructure Chem..

[203] Kalaga P.S., Kumar D., Ang D.S., Tsakadze Z. (2020). Highly transparent ITO/HfO_2_/ITO device for visible-light sensing. IEEE Access.

[204] Sivaneri K.V.I., Ozmen O., Aziziha M., Sabolsky E.M., Evans T.H., DeVallance D.B., Johnson M.B. (2019). Robust polymer-HfO_2_ thin film laminar composites for tactile sensing applications. Smart Mater. Struct..

[205] Zardetto V., Brown T.M., Reale A., Di Carlo A. (2011). Substrates for flexible electronics: A practical investigation on the electrical, film flexibility, optical, temperature, and solvent resistance properties. J. Polym. Sci. Part B Polym. Phys..

[206] Shigesato Y., Koshi-ishi R., Kawashima T., Ohsako J. (2000). Early stages of ITO deposition on glass or polymer substrates. Vacuum.

[207] Jeong J.-A., Park Y.-S., Kim H.-K. (2010). Comparison of electrical, optical, structural, and interface properties of IZO-Ag-IZO and IZO-Au-IZO multilayer electrodes for organic photovoltaics. J. Appl. Phys..

[208] Cairns D.R., Crawford G.P. (2005). Electromechanical properties of transparent conducting substrates for flexible electronic displays. Proc. IEEE.

[209] Leterrier Y., Médico L., Demarco F., Månson J.A.E., Betz U., Escolà M.F., Kharrazi Olsson M., Atamny F. (2004). Mechanical integrity of transparent conductive oxide films for flexible polymer-based displays. Thin Solid Film..

[210] Sebastian M.T., Jantunen H. (2010). Polymer–ceramic composites of 0–3 connectivity for circuits in electronics: A review. Int. J. Appl. Ceram. Technol..

[211] Meng L.-J., Placido F. (2003). Annealing effect on ITO thin films prepared by microwave-enhanced dc reactive magnetron sputtering for telecommunication applications. Surf. Coat. Technol..

[212] Seyhan A., Kartal E. (2023). Optical, electrical and structural properties of ITO/IZO and IZO/ITO multilayer transparent conductive oxide films deposited via radiofrequency magnetron sputtering. Coatings.

[213] Bandara T.M.W.J., Aththanayake A.A.A.P., Kumara G.R.A., Samarasekara P., DeSilva L.A., Tennakone K. (2021). Transparent and conductive f-doped SnO_2_ nanostructured thin films by sequential nebulizer spray pyrolysis. MRS Adv..

[214] Malik S.B., Annanouch F.E., D’Souza R., Bittencourt C., Todorović M., Llobet E. (2024). High-yield ws2 synthesis through sulfurization in custom-modified atmospheric pressure chemical vapor deposition reactor, paving the way for selective nh3 vapor detection. ACS Appl. Mater. Interfaces.

[215] Raju Nagiri R.C., Yambem S.D., Lin Q., Burn P.L., Meredith P. (2015). Room-temperature tilted-target sputtering deposition of highly transparent and low sheet resistance al doped ZnO electrodes. J. Mater. Chem. C.

[216] Giraldi T.R., Escote M.T., Bernardi M.I.B., Bouquet V., Leite E.R., Longo E., Varela J.A. (2004). Effect of thickness on the electrical and optical properties of sb doped SnO_2_ (ATO) thin films. J. Electroceramics.

[217] Ramzan M., Rana A.M., Ahmed E., Bhatti A.S., Hafeez M., Ali A., Nadeem M.Y. (2014). Optical description of HfO_2_/Al/HfO_2_ multilayer thin film devices. Curr. Appl. Phys..

[218] Hanus F., Jadin A., Laude L.D. (1996). Pulsed laser deposition of high quality ITO thin films. Appl. Surf. Sci..

[219] Zhu B.L., Liu F., Li K., Lv K., Wu J., Gan Z.H., Liu J., Zeng D.W., Xie C.S. (2017). Sputtering deposition of transparent conductive f-doped SnO_2_ (FTO) thin films in hydrogen-containing atmosphere. Ceram. Int..

[220] Park K.C., Ma D.Y., Kim K.H. (1997). The physical properties of al-doped zinc oxide films prepared by rf magnetron sputtering. Thin Solid Film..

[221] Kim W.-H., Maeng W.J., Kim M.-K., Kim H. (2011). Low pressure chemical vapor deposition of aluminum-doped zinc oxide for transparent conducting electrodes. J. Electrochem. Soc..

[222] Burunkaya E., Kiraz N., Kesmez Ö., Erdem Çamurlu H., Asiltürk M., Arpaç E. (2010). Preparation of aluminum-doped zinc oxide (AZO) nano particles by hydrothermal synthesis. J. Sol-Gel Sci. Technol..

[223] Deva Arun Kumar K., Ganesh V., Shkir M., AlFaify S., Valanarasu S. (2018). Effect of different solvents on the key structural, optical and electronic properties of sol–gel dip coated azo nanostructured thin films for optoelectronic applications. J. Mater. Sci. Mater. Electron..

[224] Wang Y., Xu G., Yang J., Mao W., Wang J., Liu Z., Dong Y., Yang S., Li J. (2022). Fabrication of AZO and FAZO films using low-cost spin-coating method. Opt. Mater..

[225] Kaid M.A., Ashour A. (2007). Preparation of ZnO-doped al films by spray pyrolysis technique. Appl. Surf. Sci..

[226] Wang B., Zhang W., Zhao F., Yu W.W., Elezzabi A.Y., Liu L., Li H. (2023). An overview of recent progress in the development of flexible electrochromic devices. Nano Mater. Sci..

[227] Baldassarri C., Shehabi A., Asdrubali F., Masanet E. (2016). Energy and emissions analysis of next generation electrochromic devices. Sol. Energy Mater. Sol. Cells.

[228] Li S., Wang Q., Zhai Y., Xing Z., Zhong J., Chao D., Zhu X., Rong C., Wu Z., Chen Z. (2023). Oligo (ethylene glycol) side chain engineering: An efficient way for boosting the development of green-solvent processable electrochromic devices. Chem. Eng. J..

[229] Kandpal S., Ezhov I., Tanwar M., Nazarov D., Olkhovskii D., Filatov L., Maximov M.Y., Kumar R. (2023). Plasma assisted atomic layer deposition NiO nanofilms for improved hybrid solid state electrochromic device. Opt. Mater..

[230] Kausar A. (2024). Thermochromic and electrochromic characteristics of graphene reinforced polymeric nanocomposites—A review. Polym. Plast. Technol. Mater..

[231] Cannavale A., Cossari P., Eperon G.E., Colella S., Fiorito F., Gigli G., Snaith H.J., Listorti A. (2016). Forthcoming perspectives of photoelectrochromic devices: A critical review. Energy Environ. Sci..

[232] Yu Z., Cai G., Liu X., Tang D. (2021). Pressure-based biosensor integrated with a flexible pressure sensor and an electrochromic device for visual detection. Anal. Chem..

[233] Yang P., Sun P., Mai W. (2016). Electrochromic energy storage devices. Mater. Today.

